# Kinetic insights into the peroxygenase activity of cellulose-active lytic polysaccharide monooxygenases (LPMOs)

**DOI:** 10.1038/s41467-020-19561-8

**Published:** 2020-11-13

**Authors:** Riin Kont, Bastien Bissaro, Vincent G. H. Eijsink, Priit Väljamäe

**Affiliations:** 1 Institute of Molecular and Cell Biology, University of Tartu, Tartu, Estonia; 2 Faculty of Chemistry, Biotechnology and Food Science, NMBU—Norwegian University of Life Sciences, Ås, Norway; 3 INRAE, Aix Marseille University, UMR1163 Biodiversité et Biotechnologie Fongiques, 13009 Marseille, France

**Keywords:** Biocatalysis, Oxidoreductases

## Abstract

Lytic polysaccharide monooxygenases (LPMOs) are widely distributed in Nature, where they catalyze the hydroxylation of glycosidic bonds in polysaccharides. Despite the importance of LPMOs in the global carbon cycle and in industrial biomass conversion, the catalytic properties of these monocopper enzymes remain enigmatic. Strikingly, there is a remarkable lack of kinetic data, likely due to a multitude of experimental challenges related to the insoluble nature of LPMO substrates, like cellulose and chitin, and to the occurrence of multiple side reactions. Here, we employed competition between well characterized reference enzymes and LPMOs for the H_2_O_2_ co-substrate to kinetically characterize LPMO-catalyzed cellulose oxidation. LPMOs of both bacterial and fungal origin showed high peroxygenase efficiencies, with *k*_cat_/*K*_mH2O2_ values in the order of 10^5^–10^6^ M^−1^ s^−1^. Besides providing crucial insight into the cellulolytic peroxygenase reaction, these results show that LPMOs belonging to multiple families and active on multiple substrates are true peroxygenases.

## Abstract (web summary)

Lytic polysaccharide monooxygenases (LPMOs) catalyze the hydroxylation of glycosidic bonds in polysaccharides, but the catalytic properties of these monocopper enzymes remain poorly characterized. Here authors employ competition between reference enzymes and LPMOs for the H_2_O_2_ co-substrate to kinetically characterize LPMO-catalyzed cellulose oxidation.

## Introduction

Lytic polysaccharide monooxygenases (LPMOs) are mono-copper enzymes involved in degradation of polysaccharides, including cellulose and chitin, which are the most abundant polysaccharides in Nature. Their oxidative mechanism was discovered in 2010 by showing that the chitin-binding protein CBP21^1,2^ of the bacterium *Serratia marcescens* (here referred to as *Sm*AA10A) catalyzes oxidative cleavage of β-1,4 glycosidic bonds in chitin, while generating C1-oxidized oligosaccharide products^3^. The reaction was shown to be dependent on the presence of O_2_ and reductant^3^. Ten years of intensive research has revealed the presence of LPMOs in most kingdoms of life^3–7^ and today, these enzymes are classified within seven families of auxiliary activities (AA)^8^ in the database of carbohydrate-active enzymes^9^, with families AA9, from fungal origin, and AA10, primarily from bacterial origin, being the best studied. To date, many LPMOs, acting on various oligo- and polysaccharides, and using different regioselectivities of oxidation, have been identified^10^.

While structurally and functionally well characterized^7,11^, kinetic studies of LPMOs are scarce. The scarcity of kinetic data likely reflects the numerous experimental challenges associated with quantitative characterization of LPMO functionality^12^. The complexity is exemplified by the recent discovery that LPMOs may use H_2_O_2_, rather than, or even instead of, O_2_, as co-substrate^13–20^. Regardless of the mechanism, LPMOs need an external electron donor for catalysis. In the H_2_O_2_-driven reaction, the reductant is used only in a “priming-reduction” of the Cu(II) resting state to the catalytically active Cu(I) form^13,18,21^ whereas in the O_2_-driven reaction the reductant is consumed in stoichiometric amounts (i.e., delivery of two electrons per glycosidic bond cleavage)^10,22^. The auto oxidation of reductants by O_2_ often leads to the formation of H_2_O_2_ and this complicates the interpretation of kinetic data. Furthermore, LPMOs that are free from substrate have both reductant oxidase^23^ and peroxidase^16,18^ activity where the oxidase activity leads to the formation of H_2_O_2_^17,23–25^.

To date only two detailed kinetic studies of LPMO action on carbohydrate substrates are available. Kuusk et al.^14^ have shown that the H_2_O_2_-driven oxidation of insoluble chitin by (bacterial) *Sm*AA10A has a *k*_cat_/${K_{{\rm{mH}}_2{\rm{O}}_2}}$ in the order of 10^6^ M^−1^ s^−1^, whereas Hangasky et al.^26^ have shown that O_2_-driven oxidation of soluble cellohexaose by an LPMO from the fungus *Myceliophthora thermophilia* (*Mt*PMO9E) has a *k*_cat_/${K_{{\rm{mO}}_2}}$ in the order of 10^3^ M^−1^ s^−1^. While the relative importance of the O_2_- and H_2_O_2_-driven reaction mechanisms remains a subject of debate^15^, available data clearly show that the peroxygenase reaction is much faster^14,18,22,27^. Unfortunately, due to a lack of suitable methods, so far, there are no solid kinetic data for one of the most important LPMO reactions, namely oxidative degradation of insoluble cellulose.

In Nature^27^ as well as in industrial applications^28–30^, LPMOs involved in lignocellulose degradation operate in redox-active environments where both potential co-substrates, O_2_ and H_2_O_2_, as well as multiple compounds and/or enzymes interacting with these co-substrates, are present. For example, the secretomes of fungi growing on lignocellulosic biomass contain, next to LPMOs, a myriad of different enzymes including several H_2_O_2_-consuming enzymes like different peroxidases and peroxygenases^27^. Comparative studies of the *k*_cat_/${K_{{\rm{mH}}_2{\rm{O}}_2}}$ values of the various H_2_O_2_-consuming enzymes in such secretomes could provide some insight into the relevance of the H_2_O_2_-driven LPMO reaction and the flow of H_2_O_2_ through different enzyme reactions. In this regard, one must bear in mind that the use of *k*_cat_/${K_{{\rm{mH}}_2{\rm{O}}_2}}$ values as performance metrics in comparing different enzymes assumes that the concentration of H_2_O_2_ is much lower than the apparent ${K_{{\rm{mH}}_2{\rm{O}}_2}}$ values of the competing enzymes^31^.

Here, inspired by the work of Wang et al.^32^ who demonstrated in 2013 that the fosfomycin-producing epoxidase HppE is a non-heme-iron peroxidase rather than an oxidase, we have developed a theoretical framework and an experimental setup to kinetically characterize a so far non-characterized and very important LPMO reaction, the cellulolytic peroxygenase reaction. We have addressed this issue by setting up competition experiments with a kinetically well characterized reference enzyme and an LPMO of interest, to derive *k*_cat_/${K_{{\rm{mH}}_2{\rm{O}}_2}}$ values for LPMOs acting on soluble cellooligosaccharides as well as insoluble cellulose (Avicel). One of the two reference enzymes in this study was a chitin-active bacterial LPMO, *Sm*AA10A, for which detailed kinetic data exist thanks to the availability of unique, but not generally available, experimental tools that include the use of ^14^C-labeled chitin nanowhiskers (CNWs)^14^. The other reference enzyme was readily available horseradish peroxidase (HRP) acting on two different, both readily available, substrates, *N*-acetyl-3,7-dihydroxyphenoxazine (Amplex Red, AR) and 2,2′-azino-bis(3-ethylbenzothiazoline-6-sulfonic acid) diammonium salt (ABTS). The cellulose-active LPMOs studied were fungal family AA9 LPMOs from *Trichoderma reesei* (*Tr*AA9A) and *Neurospora crassa* (*Nc*AA9C), as well as a bacterial AA10 LPMO from *Streptomyces coelicolor* (*Sc*AA10C, formerly also known as CelS2).

## Results

### Two enzymes competing for the same co-substrate—kinetic foundation

**Fig. 1:**
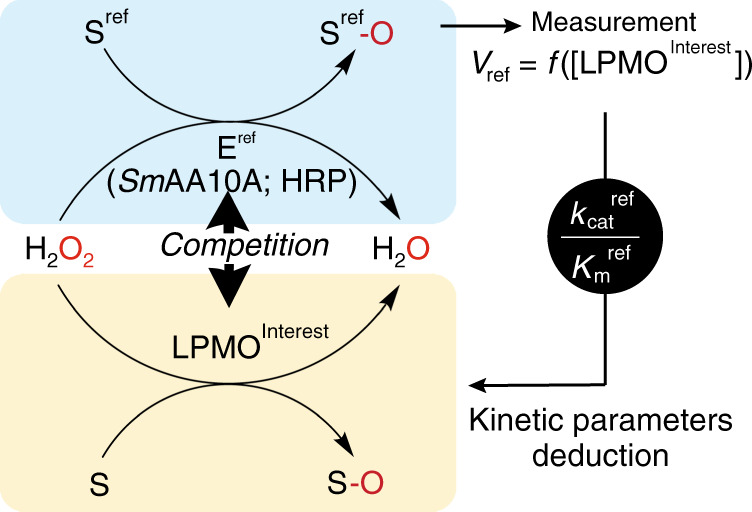
Two enzymes, a reference enzyme (E^ref^, i.e., *Sm*AA10A or HRP) and an LPMO of interest compete for the H_2_O_2_ co-substrate. The rate of the reference reaction (*v*_ref_) is a function of the concentration of the LPMO of interest. Provided with the *k*_cat_/*K*_m_ (for H_2_O_2_) value for reference reaction one can calculate the *k*_cat_/*K*_m_ (for H_2_O_2_) value for the LPMO reaction (oxidation of substrate, S). Note that, in the case of HRP, the reference substrate (S^ref^) is oxidized but the oxygen atom is not incorporated into S^ref^.

Let us consider a system where two enzymes, the reference enzyme and the enzyme of interest compete for the H_2_O_2_ co-substrate (Fig. 1). The substrate of the reference enzyme is designated as S^ref^ and that of the enzyme of interest as S. The experimental approach used in this study requires that the values of the kinetic parameters of the reference enzyme are known. To this end, we used the well-characterized chitin-active *Sm*AA10A as well as HRP as reference enzymes (see below).

Enzyme reactions involving two substrates may follow a ternary complex (both substrates are bound to the enzyme) or a ping-pong mechanism. Depending on the order of the binding of substrates the mechanisms involving a ternary complex are further grouped as random order or ordered mechanisms. In the absence of products, the steady-state rates of H_2_O_2_ consumption (*d*[H_2_O_2_]/*dt*) for an enzyme obeying an ordered (with S being the first substrate to bind) ternary complex or a ping-pong mechanism are given by Eqs. (1) and (2), respectively^33,34^.

1 $$v_{\rm{ref}} 	= - \frac{{d\left[ {{{\rm{H}}_2{\rm{O}}_2}} \right]}}{{dt}} \\ 	= \frac{{k_{\rm{cat}}^{{\rm{ref}}}\left[ {{\rm{E}}^{\rm{ref}}} \right]\left[ {{{\rm{H}}_2{\rm{O}}_2}} \right]\left[ {{\rm{S}}^{\rm{ref}}} \right]}}{{K_{\rm{iS}}^{\rm{ref}}K_{{{{\rm{mH}}_2{\rm{O}}_2}}}^{\rm{ref}} + K_{{\rm{mS}}}^{\rm{ref}}\left[ {{{\rm{H}}_2{\rm{O}}_2}} \right] + K_{{{{\rm{mH}}_2{\rm{O}}_2}}}^{\rm{ref}}\left[ {\rm{S}^{\rm{ref}}} \right] + \left[ {{{\rm{H}}_2{\rm{O}}_2}} \right]\left[ {\rm{S}^{\rm{ref}}} \right]}},$$

2 $$v_{\rm{ref}} = - \frac{{d\left[ {{{\rm{H}}_2{\rm{O}}_2}} \right]}}{{dt}} = \frac{{k_{\rm{cat}}^{\rm{ref}}\left[ {E^{\rm{ref}}} \right]\left[ {{{\rm{H}}_2{\rm{O}}_2}} \right]\left[ {\rm{S}^{\rm{ref}}} \right]}}{{K_{\rm{mS}}^{\rm{ref}}\left[ {{{\rm{H}}_2{\rm{O}}_2}} \right] + K_{{{{\rm{mH}}_2{\rm{O}}_2}}}^{\rm{ref}}\left[ {\rm{S}^{\rm{ref}}} \right] + \left[ {{{\rm{H}}_2{\rm{O}}_2}} \right]\left[ {\rm{S}^{\rm{ref}}} \right]}}.$$

The superscript ref refers to the reference enzyme but the same equations apply for the enzyme of interest (in this case we do not use superscript ref). [H_2_O_2_], [S] and [E] stand for the concentration of H_2_O_2_, substrate, and the total concentration of enzyme, respectively. *k*_cat_ is the catalytic constant (based on H_2_O_2_ consumption) and ${K_{{\rm{mH}}_2{\rm{O}}_2}}$ and *K*_mS_ are the Michaelis constants for H_2_O_2_ and substrate, respectively. *K*_iS_ is “inhibition” constant for substrate (i.e., binding constant of S in the absence of H_2_O_2_)^33^. In its general form, Eq. (1) also applies to an ordered mechanism with H_2_O_2_ being the first substrate to bind (Supplementary discussion, Eq. (S1)). Assuming that binding reactions are at equilibrium, Eq. (1) also applies to the random order mechanism (Supplementary discussion, Eq. (S2)).

In the competition experiments described herein below, both the reference enzyme and the enzyme of interest are present simultaneously, but only the turnover of S^ref^, resulting from an enzyme acting with a rate of *v*_ref_ is followed (Fig. 1). The ratio between *v*_ref_ and the rate of the enzyme of interest, *v*, can be obtained using Eqs. (1) and (2). For straightforward interpretation of *v*_ref_/*v* ratios we use two simplifying assumptions. (i) [H_2_O_2_] ≪ ${K_{{\rm{mH}}_2{\rm{O}}_2}}$^ref^ and (ii) [H_2_O_2_] ≪ ${K_{{\rm{mH}}_2{\rm{O}}_2}}$. In this case, the kinetics of both enzymes is governed by the apparent *k*_cat_/${K_{{\rm{mH}}_2{\rm{O}}_2}}$ (Supplementary discussion, Eqs. (S3–S8)). Within these constraints and assuming a ternary complex mechanism for the enzyme of interest, the *v*_ref_/*v* is given by Eq. (3) (for E^ref^ obeying an ordered ternary complex mechanism, such as for *Sm*AA10A^14,18^) or Eq. (4) (for E^ref^ obeying a ping-pong mechanism, such as for HRP^35^) (see supplementary “kinetic foundation” for more details).

3 $$\frac{{v_{\rm{ref}}}}{v} = \frac{{\frac{{k_{\rm{cat}}^{\rm{ref}}}}{{K_{m{{{\rm{H}}_2{\rm{O}}_2}}}^{\rm{ref}}}}\left[ {\rm{E}^{\rm{ref}}} \right]\left( {\frac{{\left[ {\rm{S}^{\rm{ref}}} \right]}}{{\left[ {\rm{S}^{\rm{ref}}} \right] + K_{\rm{iS}}^{\rm{ref}}}}} \right)}}{{\frac{{k_{\rm{cat}}}}{{K_{m{{{\rm{H}}_2{\rm{O}}_2}}}}}\left[ {\rm{E}} \right]\left( {\frac{{\left[ {\rm{S}} \right]}}{{\left[ {\rm{S}} \right] + \left[ {\rm{S}} \right]_{0.5}}}} \right)}},$$

4 $$\frac{{v_{\rm{ref}}}}{v} = \frac{{\frac{{k_{\rm{cat}}^{\rm{ref}}}}{{K_{m{{{\rm{H}}_2{\rm{O}}_2}}}^{ref}}}\left[ {\rm{E}^{ref}} \right]}}{{\frac{{k_{\rm{cat}}}}{{K_{m{{{\rm{H}}_2{\rm{O}}_2}}}}}\left[ \rm{E} \right]\left( {\frac{{\left[ \rm{S} \right]}}{{\left[ \rm{S} \right] + \left[ \rm{S} \right]_{0.5}}}} \right)}}.$$

Since the exact interpretation of the meaning of the binding constant (*K*_iS_) depends on the order of the binding of substrates (Supplementary discussion, Eqs. (S6) and (S7)), which is not known for the LPMOs of interest, we have replaced this parameter with the empirical constant [S]_0.5_.

Let’s define the concentration of the enzyme of interest ([E]) that halves the rate of the reference reaction (i.e., *v* = *v*_ref_) as [E]_50_. The [E]_50_ can be found by measuring *v*_ref_ in experiments with different [E] and by analyzing the data according to Eq. (5).

5 $$\frac{{v_{\rm{ref}}}}{{v_{\rm{ref}} + v}} = \frac{{v_{\rm{ref}}}}{{V_{\rm{lim}}}} = \frac{1}{{1 + \frac{{\left[ \rm{E} \right]}}{{\left[ \rm{E} \right]_{50}}}}}.$$

The term *V*_lim_ in Eq. (5) refers to the maximum, and limiting, rate of the reaction (in experiments *V*_lim_ is measured as *v*_ref_ in the absence of competing enzyme). Provided with the value of [E]_50_ and the values of the parameters of the reference reaction, the apparent *k*_cat_/${K_{{\rm{mH}}_2{\rm{O}}_2}}$ for the enzyme of interest can be calculated using Eq. (6).

6 $$\left( {\frac{{k_{\rm{cat}}}}{{K_{{{\rm{mH}}_2{\rm{O}}_2}}}}} \right)^{\rm{app}} = \left( {\frac{{k_{\rm{cat}}^{\rm{ref}}}}{{K_{{{\rm{mH}}_2{\rm{O}}_2}}^{\rm{ref}}}}} \right)^{\rm{app}}\left( {\frac{{\left[ {\rm{E}^{ref}} \right]}}{{\left[ \rm{E} \right]_{50}}}} \right).$$

Finally, the true *k*_cat_/${K_{{\rm{mH}}_2{\rm{O}}_2}}$ value can be found from the analysis of (*k*_cat_/${K_{{\rm{mH}}_2{\rm{O}}_2}}$)^app^ values measured at different [S], according to Eq. (7).

7 $$\left( {\frac{{k_{\rm{cat}}}}{{K_{{{\rm{mH}}_2{\rm{O}}_2}}}}} \right)^{\rm{app}} = \frac{{\left( {\frac{{k_{\rm{cat}}}}{{K_{{{\rm{mH}}_2{\rm{O}}_2}}}}} \right)\left[ \rm{S} \right]}}{{\left[ \rm{S} \right]_{0.5} + \left[ \rm{S} \right]}}.$$

### Development of the experimental method: proof-of-concept

As a proof-of-concept, we first carried out competition experiments with two reference enzymes with known kinetic parameters, namely *Sm*AA10A and HRP. The kinetics of H_2_O_2_-driven degradation of CNWs by *Sm*AA10A has been characterized in detail before^14,21^. Provided with ascorbic acid (0.1 mM) as reductant, *Sm*AA10A has a *k*_cat_ value for H_2_O_2_-driven oxidative cleavage of CNWs (*c.f*. consumption of H_2_O_2_) of 6.7 ± 0.2 s^−1^. The *K*_iS_ value for CNWs is 0.68 ± 0.01 g L^−1^, and the *K*_m_ values are 2.8 ± 1.3 µM and 0.58 ± 0.05 g L^−1^ for H_2_O_2_ and CNWs, respectively^14^. The *Sm*AA10A has been proposed to obey an ordered ternary complex mechanism with CNWs being the first substrate to bind^14^. We chose HRP as the second H_2_O_2_ consuming reference enzyme. HRP is a heme-peroxidase that obeys a ping-pong mechanism^35^. Since *Sm*AA10A has previously been characterized at pH 6.1 and 25 °C^14^, the same experimental conditions were used throughout this study. When using ABTS as substrate, we measured a *k*_cat_/*K*_m_ value for H_2_O_2_ of 7.1 ± 1.2 µM^−1^ s^−1^ (Supplementary Fig. 1). Although both the *k*_cat_ and *K*_m_ for H_2_O_2_ increased with increasing [ABTS], the *k*_cat_/*K*_m_ was independent of [ABTS] (Supplementary Fig. 1). This kinetic signature is characteristic for the ping-pong mechanism.

Because of the [H_2_O_2_] ≪ ${K_{{\rm{mH}}_2{\rm{O}}_2}}$ constraint, introduced to simplify the equations, and the low ${K_{{\rm{mH}}_2{\rm{O}}_2}}$ for *Sm*AA10A, the measurement of initial rates at fixed H_2_O_2_ load was not applicable in this study. An alternative is in situ generation of H_2_O_2_ at constant rate, e.g., by addition of glucose oxidase and its substrate, which may lead to sufficiently low steady-state conditions. The rate of H_2_O_2_ generation sets limits to the maximum rate of its consumption and for this reason we refer to it as the limiting rate (*V*_lim_). In the steady-state, *v*_ref_ + *v* = *V*_lim_. Importantly, besides providing LPMOs with electrons, AscA, a commonly used reductant in LPMO research, can also be subject to auto-oxidation by O_2_ leading to the formation of H_2_O_2_^36^. Furthermore, H_2_O_2_ is also produced in uncoupled reactions of reduced LPMOs with O_2_^17,23,24^. For the simplicity of data interpretation, it was important to use reaction conditions in which auto-oxidation of AscA and LPMO-mediated production of H_2_O_2_ are insignificant, in other words, conditions in which *V*_lim_ is independent of [LPMO]. It was also important to verify that the substrates for the enzymes of interest do not inhibit the reference enzyme and vice versa, i.e the substrate of the reference enzyme shall not inhibit the enzyme of interest.

To ensure that we met these conditions, we analyzed the inhibition of *Sm*AA10A-catalyzed oxidation of CNWs by HRP/ABTS systems with auto-oxidation of AscA (1.0 mM) as a source of H_2_O_2_. Of note, while H_2_O_2_ can directly react with AscA this reaction is slow^37^ and can be considered insignificant compared to H_2_O_2_-driven enzyme reactions. As initial controls, we first showed that the rate of formation of ^14^C-soluble products (expressed in NAG equivalents, NAG_eq_) in absence of competing enzyme, i.e., *V*_lim_, was independent on [*Sm*AA10A] (Supplementary Fig. 2) and thus limited by the in situ production of H_2_O_2_ originating from AscA auto-oxidation. This shows that the production of H_2_O_2_ by *Sm*AA10A is negligible. The addition of 0.2 mM ABTS had no effect on the steady-state rate of NAG_eq_ formation (Supplementary Fig. 2). However, since the *V*_lim_ is independent on [*Sm*AA10A] (Supplementary Fig. 2), inhibition of *Sm*AA10A may not be revealed in this experimental set-up^29^. Therefore, the inhibition must be assessed by measuring effects on the initial rates at fixed H_2_O_2_ concentration. Reactions that were set up to this end, using 0.1 mM AscA as reductant, CNWs (1.0 g L^−1^) and 20 µM H_2_O_2_ as co-substrate, showed that ABTS as well as another HRP substrate, AR, had a small inhibiting effect on *Sm*AA10A (Supplementary Fig. 3). Regarding the potential cross-effects on the reference systems (*Sm*AA10A/CNW or HRP/ABTS) of “cellulosic” substrates (Glc_5_ and Avicel) used by the LPMOs of interest that are discussed below, no significant inhibitory effect on the *Sm*AA10A/CNW system was observed (Supplementary Fig. 3). For technical reasons, similar control experiments for the HRP/ABTS system were not possible, but the experiments described below indicate that also in this case interference by cellulose substrates is minimal.

**Fig. 2:**
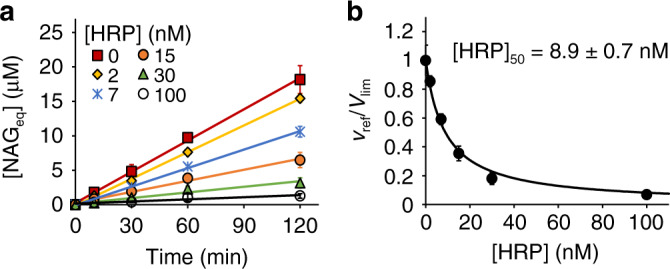
Competition between *Sm*AA10A and HRP. All experiments were made in Bis–Tris buffer (50 mM, pH 6.1) at 25 °C and contained CNWs (1.0 g L^−1^), AscA (1.0 mM), *Sm*AA10A (42 nM), and ABTS (0.2 mM). **a** Progress curves for the release of soluble products (in NAG_eq_) in the presence of horseradish peroxidase (HRP) at different concentrations, as indicated in the plot. Solid lines show linear regression of the data. The slopes of the lines correspond to the rate of the reference reaction (*v*_ref_). **b** Dependency of *v*_ref_/*V*_lim_ on [HRP]. The solid line shows non-linear regression of the data according to Eq. (5). Data are presented as average values (*n* = 3, independent experiments) and error bars show SD. Source data are provided as a Source Data file.

A competition experiment using the conditions verified above showed that the presence of HRP, here treated as the enzyme of interest, decreased the steady-state rates of the *Sm*AA10A reaction, the reference reaction, in an [HRP] dependent manner (Fig. 2a). Analysis of the dependency of *v*_ref_/*V*_lim_ on [HRP] (Fig. 2b) according to Eq. (5) resulted in an [HRP]_50_ value of 8.9 ± 0.7 nM. Based on the known kinetic parameters^14^ and [CNW] = 1.0 g L^−1^, one can calculate a (*k*_cat_/${K_{{\rm{mH}}_2{\rm{O}}_2}}$)^app^ value of 1.5 ± 0.67 µM^−1^ s^−1^ for *Sm*AA10A. Inserting this figure along with [CBP21] = 42 nM and the measured value of [HRP]_50_ into Eq. (6) results in a *k*_cat_/${K_{{\rm{mH}}_2{\rm{O}}_2}}$ value of 7.1 ± 3.3 µM^−1^ s^−1^ for HRP (since HRP obeys ping-pong mechanism, the analysis by Eq. (6) gives a true *k*_cat_/*K*_m_). This figure is on par with the *k*_cat_/*K*_m_ value of 7.1 ± 1.2 µM^−1^ s^−1^ obtained from direct measurement of the oxidation of ABTS by HRP (Supplementary Fig. 1). Importantly, the results of this proof-of-concept experiment show that the combination of the experimental set-up and kinetic foundation described above allows the generation of meaningful kinetic data for the enzyme of interest.

### Measuring *k*_cat_/${K_{{\rm{mH}}_2{\rm{O}}_2}}$ values for fungal cellulose-active LPMOs using the *Sm*AA10A/CNW reference reaction

**Fig. 3:**
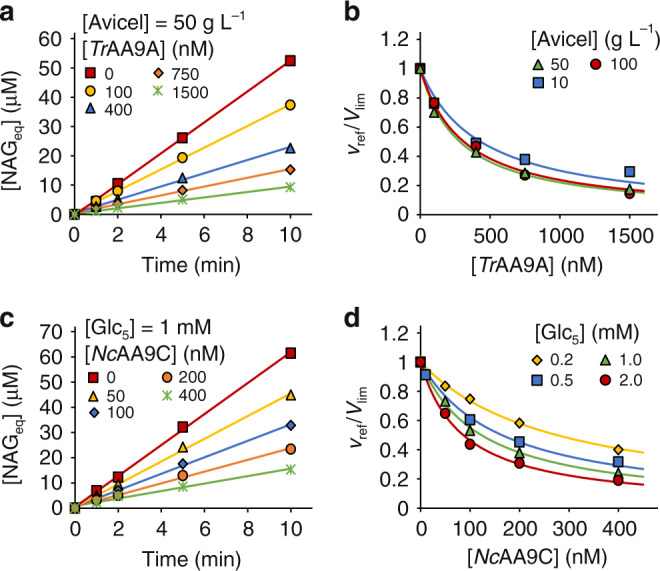
Inhibition of *Sm*AA10A/CNW by *Tr*AA9A/Avicel and *Nc*AA9C/Glc_5_. All experiments were made in Bis–Tris buffer (50 mM, pH 6.1) at 25 °C and contained CNWs (1.0 g L^−1^), AscA (0.1 mM), *Sm*AA10A (50 nM), GO (0.03 g L^−1^), and glucose (10 mM). **a** Progress curves for the release of soluble products (in NAG_eq_) in the presence of *Tr*AA9A at different concentrations and 50 g L^−1^ Avicel (for progress curves with other [Avicel], see Supplementary Fig. 4). Solid lines show linear regression of the data. The slopes of the lines correspond to the rate of the reference reaction (*v*_ref_). **b** Dependency of *v*_ref_/*V*_lim_ on [*Tr*AA9A], at different Avicel concentrations. Solid lines show nonlinear regression of the data according to Eq. (5). **c** Progress curves for the release of soluble products (in NAG_eq_) in the presence of *Nc*AA9C at different concentrations and 1.0 mM Glc_5_ (for progress curves with other [Glc_5_], see Supplementary Fig. 6). Solid lines show linear regression of the data. The slopes of the lines correspond to the rate of reference reaction (*v*_ref_). **d** Dependency of *v*_ref_/*V*_lim_ on [*Nc*AA9C] at different [Glc_5_]. Solid lines show nonlinear regression of the data according to Eq. (5). Data are presented as average values (*n* = 2, independent experiments). Source data are provided as a Source Data file.

**Fig. 4:**
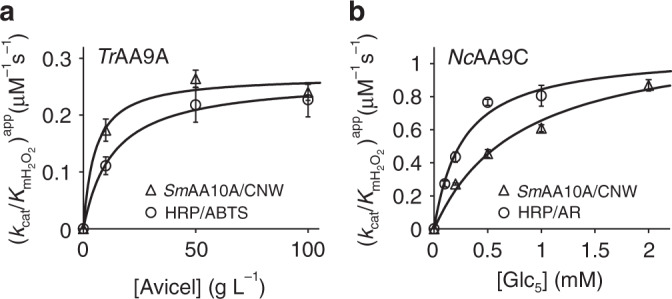
Apparent *k*_cat_/${K_{{\rm{mH}}_2{\rm{O}}_2}}$ values for *Tr*AA9A/Avicel and *Nc*AA9C/Glc5 measured using different reference reactions. **a** Apparent *k*_cat_/${K_{{\rm{mH}}_2{\rm{O}}_2}}$ values for *Tr*AA9A/Avicel measured using the *Sm*AA10A/CNW or the HRP/ABTS reference reaction. **b** Apparent *k*_cat_/${K_{{\rm{mH}}_2{\rm{O}}_2}}$ values for *Nc*AA9C/Glc_5_ measured using the *Sm*AA10A/CNW or the HRP/AR reference reaction. Solid lines show nonlinear regression of the data according to Eq. (7). Apparent *k*_cat_/${K_{{\rm{mH}}_2{\rm{O}}_2}}$ values and their errors (SD) were calculated from corresponding [*E*]_50_ values and their associated errors that were derived from nonlinear regression analysis (for details see Source Data file).

Here we assessed the inhibition of *Sm*AA10A/CNW by AA9 family LPMOs, either from *T. reesei* (*Tr*AA9A, formerly *Tr*Cel61A) acting on microcrystalline cellulose (Avicel), or from *N. crassa* (*Nc*AA9C) acting on soluble cello-oligosaccharides such as cellopentaose (Glc_5_). AscA (0.1 mM) was added to ensure priming reduction of the LPMOs. To obtain a high *V*_lim_ the H_2_O_2_ was generated using the glucose oxidase (GO) reaction (10 mM glucose). A high *V*_lim_ is important, since it ensures that generation of H_2_O_2_ by auto-oxidation of AscA (present at low concentration) or by uncoupled reaction of reduced LPMOs with O_2_ becomes insignificant. Figure 3a and Supplementary Fig. 4 show the progress curves of NAG_eq_ formation by *Sm*AA10A in the presence of Avicel and different concentrations of *Tr*AA9A. In all cases the release of NAG_eq_ in time was linear and the slope of the lines was used to measure the steady-state rates of the *Sm*AA10A reaction. The strength of the inhibition of *Sm*AA10A increased with increasing [*Tr*AA9A] and [Avicel] (Fig. 3b). Of note, neither Avicel (Supplementary Figs. 3 and 5a) nor Glc_5_ (Supplementary Figs. 3 and 5b) inhibited the *Sm*AA10A/CNW system (in the absence of AA9). The apparent *k*_cat_/${K_{{\rm{mH}}_2{\rm{O}}_2}}$ values for *Tr*AA9A/Avicel were calculated from the values of half-inhibiting concentrations of *Tr*AA9A ([*Tr*AA9A]_0.5_) according to Eq. (6) using the known parameter values for the *Sm*AA10A/CNW reference system. [*Tr*AA9A]_0.5_ decreased while (*k*_cat_/${K_{{\rm{mH}}_2{\rm{O}}_2}}$)^app^ increased with increasing [Avicel] (Table 1). The true *k*_cat_/${K_{{\rm{mH}}_2{\rm{O}}_2}}$ value of 0.27 ± 0.02 µM^−1^ s^−1^ and a half-saturating Avicel concentration of 5.2 ± 2.2 g L^−1^ ([S]_0.5_) were estimated from the dependency of (*k*_cat_/${K_{{\rm{mH}}_2{\rm{O}}_2}}$)^app^ on [Avicel] (Fig. 4a), according to Eq. (7). Similarly, we found that the strength of the inhibition of the *Sm*AA10A/CNW reaction by the *Nc*AA9C/Glc_5_ system depended on the concentrations of *Nc*LPMO9C and Glc_5_ (Fig. 3c, d, Table 1, Supplementary Fig. 6). Analysis of the (*k*_cat_/${K_{{\rm{mH}}_2{\rm{O}}_2}}$)^app^ versus [Glc_5_] curve (Fig. 4b) according to Eq. (7) suggested a true *k*_cat_/${K_{{\rm{mH}}_2{\rm{O}}_2}}$ value of 1.19 ± 0.11 µM^−1^ s^−1^ and a [S]_0.5_ for Glc_5_ of 0.81 ± 0.18 mM.

**Table 1:** Kinetic parameters of cellulose-active LPMOs measured using different reference reactions.

Reference reaction^a^	E and S of interest	[S]	[E]_50_ (nM)^b^	(*k*_cat_/*K*_m_)^app^ for H_2_O_2_ (µM^−1^ s^−1^)^c^
50 nM *Sm*AA10A, 1.0 g L^−1^ CNW	*Tr*AA9A, Avicel	10 g L^−1^	432 ± 48	0.17 ± 0.02
		50 g L^−1^	284 ± 16	0.26 ± 0.01
		100 g L^−1^	312 ± 18	0.24 ± 0.01
			True *k*_cat_/*K*_m_ = 0.27 ± 0.02 µM^−1^ s^−1 d^	
5 nM HRP, 0.2 mM ABTS	*Tr*AA9A, Avicel	10 g L^−1^	321 ± 45	0.11 ± 0.02
		50 g L^−1^	163 ± 23	0.22 ± 0.03
		100 g L^−1^	156 ± 21	0.23 ± 0.03
1 nM HRP, 0.2 mM ABTS		100 g L^−1^	35 ± 5	0.21 ± 0.03
			True *k*_cat_/*K*_m_ = 0.26 ± 0.01 µM^−1^ s^−1 d^	
50 nM *Sm*AA10A, 1.0 g L^−1^ CNW	*Nc*AA9C, Glc_5_	0.2 mM	276 ± 6	0.27 ± 0.01
		0.5 mM	165 ± 9	0.46 ± 0.02
		1.0 mM	123 ± 4	0.61 ± 0.02
		2.0 mM	86 ± 3	0.87 ± 0.03
			True *k*_cat_/*K*_m_ = 1.19 ± 0.11 µM^−1^ s^−1 d^	
5 nM HRP, 0.2 mM AmplexRed	*Nc*AA9C, Glc_5_	0.1 mM	246 ± 16	0.27 ± 0.02
		0.2 mM	154 ± 11	0.43 ± 0.03
		0.5 mM	87 ± 2	0.77 ± 0.02
		1.0 mM	83 ± 7	0.81 ± 0.06
			True *k*_cat_/*K*_m_ = 1.07 ± 0.10 µM^−1^ s^−1 d^	
10 nM *Sm*AA10A, 1.0 g L^−1^ CNW	*Sc*AA10C, Avicel	100 g L^−1^	101 ± 16	0.15 ± 0.02
1 nM HRP, 0.2 mM ABTS	*Sc*AA10C, Avicel	100 g L^−1^	57 ± 7	0.13 ± 0.02

^a^All reactions were made in 50 mM Bis–Tris buffer pH 6.1 at 25 °C and contained glucose oxidase, 10 mM glucose and 0.1 mM ascorbic acid.

^b^Half-inhibiting concentrations of the enzyme of interest were calculated from the measured rates of the reference reaction in the absence (*V*_lim_) and presence (*v*_ref_) of the enzyme of interest, according to Eq. (5).

^c^(*k*_cat_/*K*_m_)^app^ for the enzyme of interest were calculated from the concentration of the reference enzyme, the [E]_50_ and the (*k*_cat_/*K*_m_)^app^ of the reference enzyme for H_2_O_2_, using Eq. (6). The (*k*_cat_/*K*_m_)^app^ values of the reference enzymes used in calculations were 1.5 ± 0.7, 7.1 ± 1.2, and 13.4 ± 0.5 µM^−1^ s^−1^ for the *Sm*AA10A/CNW, HRP/ABTS, and HRP/AmplexRed reference reactions, respectively. The SD is for the measurement of (*k*_cat_/*K*_m_)^app^ of the enzyme of interest and does not include the SD of (*k*_cat_/*K*_m_)^app^ of the reference enzyme.

^d^The true *k*_cat_/*K*_m_ values for H_2_O_2_ were found from nonlinear regression analysis of the dependency of (*k*_cat_/*K*_m_)^app^ on [S] according to Eq. (7).

### Measuring the *k*_cat_/${K_{{\rm{mH}}_2{\rm{O}}_2}}$ values of fungal cellulose-active LPMO using the HRP/Amplex Red reference reaction

Since ^14^C labeled CNWs are not commercially available we next tested the possibility to use H_2_O_2_-driven oxidation of Amplex Red (AR) by HRP as a broadly available, easy-to-implement reference reaction system. One electron oxidation of AR by HRP results in the formation of an AR radical. Two AR radicals combine in an HRP-independent reaction to produce one molecule of AR and one molecule of resorufin, and the latter can be detected by the absorbance or fluorescence^38^. The kinetics of HRP with the AR substrate was consistent with the ping-pong mechanism and the *k*_cat_/*K*_m_ value for H_2_O_2_ consumption was determined to be 13.4 ± 0.5 µM^−1^ s^−1^ (Supplementary Fig. 7). Of note, the presence of 0.1 mM AscA (needed in the LPMO reactions) resulted in a 3-fold reduction in the rate of resorufin formation (Supplementary Fig. 8). This phenomenon can be accounted for by the reduction of the AR radical to AR by AscA^38^. This will reduce the resorufin yield but should not affect the rate of H_2_O_2_ consumption in the HRP reaction. However, to quantify the rate of HRP, a correction of the extinction coefficient was needed, as outlined in Supplementary Table 1 and Supplementary Fig. 8. *Nc*AA9C with Glc_5_ as substrate was used as the reaction of interest and the GO reaction was used as a source of H_2_O_2_. Glucose and Glc_5_ had no effect on the oxidation of AR by HRP (Supplementary Fig. 7a, b).

**Fig. 5:**
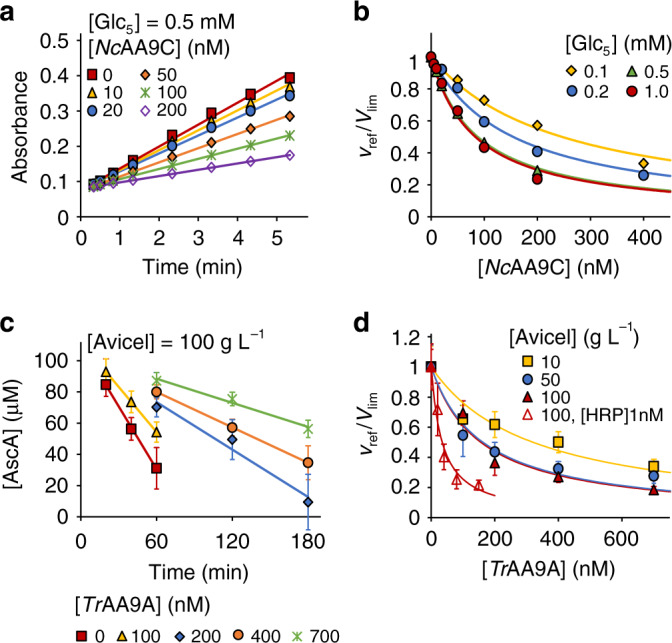
Inhibition of HRP by *Nc*AA9C/Glc_5_ and *Tr*AA9A/Avicel. All experiments were made in Bis–Tris buffer (50 mM, pH 6.1) at 25 °C and contained AscA (0.1 mM), HRP (5.0 nM), and glucose (10 mM). **a** Progress curves for the oxidation of AR (0.2 mM) (measured as a change in absorbance at 570 nm) in the presence of *Nc*AA9C at different concentrations. Reaction mixtures contained GO (0.15 g L^−1^) and Glc_5_ (0.5 mM); for progress curves for reactions with other [Glc_5_], see Supplementary Fig. 9. Solid lines show linear regression of the data. The slopes of the lines correspond to the rate of the reference reaction (*v*_ref_). **b** Dependency of *v*_ref_/*V*_lim_ on [*Nc*AA9C] at different substrate concentrations. Solid lines show non-linear regression of the data according to Eq. (5). **c** Progress curves for the oxidation of ABTS (0.2 mM) (measured as consumption of AscA) in the presence of *Tr*AA9A at different concentrations. Reaction mixtures contained GO (0.035 g L^−1^) and Avicel (100 g L^−1^); for progress curves for reactions with other [Avicel], see Supplementary Fig. 12. Solid lines show linear regression of the data. The slopes of the lines correspond to the rate of the reference reaction (*v*_ref_). **d** Dependency of *v*_ref_/*V*_lim_ on [*Tr*AA9A] at different substrate concentrations. Note that in one series (opened red triangles) the [Avicel] was 100 g L^−1^ but the [HRP] was 1.0 nM instead of the usual 5.0 nM. Solid lines show non-linear regression of the data according to Eq. (5). Data are presented as average values (*n* = 2 for **a** and **b**, *n* = 3 for **c** and **d**, independent experiments) and error bars (in **c** and **d**) show SD. Source data are provided as a Source Data file.

Studies of the inhibition of the HRP/AR reaction by *Nc*AA9C/Glc_5_ (Fig. 5a, b, Table 1) yielded results that were consistent with Eq. (5), and further analysis of the data according to Eqs. (6) and (7), as described above, yielded a true *k*_cat_/${K_{{\rm{mH}}_2{\rm{O}}_2}}$ value of 1.07 ± 0.1 µM^−1^ s^−1^ and a [S]_0.5_ for Glc_5_ of 0.23 ± 0.07 mM (Fig. 4b). Note that, while the value of *k*_cat_/ ${K_{{\rm{mH}}_2{\rm{O}}_2}}$ is well in line with the value obtained using the *Sm*AA10A/CNW reference reaction (see above; summarized in Table 1), the [S]_0.5_ values are significantly different.

### Measuring the *k*_cat_/${K_{{\rm{mH}}_2{\rm{O}}_2}}$ values of fungal cellulose-active LPMO using the HRP/ABTS reference reaction

Since we did not manage to obtain linear progress curves in HRP/AR reactions that contained insoluble cellulose, we turned to ABTS as an alternative substrate for HRP. HRP catalyzed oxidation of ABTS results in the formation of an ABTS cation radical (ABTS^•+^) that can be detected by the absorbance at 420 nm. The ABTS^•+^ radical immediately reacts with AscA present in the LPMO reaction to form ABTS and an AscA radical^37^. Therefore, the formation of ABTS^•+^ as a product of the reference reaction cannot be detected. Indeed, control experiments in which the HRP/ABTS reaction was carried out in the presence of AscA showed that there was no increase in the absorbance at 420 nm until AscA was depleted (Supplementary Fig. 10). Nevertheless, the HRP catalyzed oxidation of ABTS can be measured by measuring the rate of the depletion of AscA, which was done using a method inspired by Kracher et al.^39^, as described in the “Methods” section. Here, *Tr*AA9A-catalyzed oxidation of Avicel was the reaction of interest and GO with glucose was used as a source of H_2_O_2_. In order to ensure that the depletion of AscA could be used as a reporter of ABTS oxidation by HRP, we carried out control reactions with all reaction components except HRP (i.e., *Tr*AA9A/Avicel, GO/glucose, ABTS and 0.1 mM AscA; Supplementary Fig. 11). This experiment showed that, at high Avicel concentrations (100 g L^−1^), AscA was barely consumed, indicating that auto-oxidation of AscA was not significant under our experimental conditions, and that AscA consumption by the LPMO was only in “priming” amounts. However, some depletion of AscA occurred at lower Avicel concentrations (10 g L^−1^), from 100 µM to 77 ± 7 µM upon incubation for 3 h (Supplementary Fig. 11). This reflects the occurrence of uncoupled reactions between O_2_/H_2_O_2_ and polysaccharide-free reduced *Tr*AA9A molecules, which are expected to be more abundant at lower polysaccharide concentrations. Additional control reactions showed that, in the absence of the LPMO, the concentration of Avicel had no effect on the steady-state rate of AscA depletion in the HRP/ABTS reaction (Supplementary Fig. 5d), showing that the presence of Avicel has no effect on *V*_lim_.

The time curves of AscA depletion were linear (Fig. 5c, Supplementary Fig. 12) and the slopes were used to calculate the rate of ABTS oxidation and thus that of H_2_O_2_ consumption in the HRP reaction (*v*_ref_). H_2_O_2_ consumption in the HRP reaction was inhibited by *Tr*AA9A/Avicel in a concentration dependent manner (Fig. 5d, Table 1). Analysis of the dependency of the (*k*_cat_/${K_{{\rm{mH}}_2{\rm{O}}_2}}$)^app^ value on [Avicel] (Fig. 4a), according to Eqs. (6) and (7) gave a true *k*_cat_/${K_{{\rm{mH}}_2{\rm{O}}_2}}$ value of 0.26 ± 0.01 µM^−1^ s^−1^ and a [S]_0.5_ for Avicel of 13 ± 3.3 g L^−1^. The *k*_cat_/${K_{{\rm{mH}}_2{\rm{O}}_2}}$ is well in line with the value measured using *Sm*AA10A/CNW reference reaction (0.27 ± 0.02 µM^−1^ s^−1^; Table 1), whereas the [S]_0.5_ values differ significantly.

Since rates scale linearly with total enzyme concentration (Eqs. (1) and (2)), the measured [E]_50_ shall also scale linearly with [E]_ref_. To test the latter prediction we also measured inhibition of the HRP/ABTS reaction by *Tr*AA9A/Avicel using 100 g L^−1^ Avicel but 5-fold lower [HRP] (1.0 nM) (Fig. 5d, Supplementary Fig. 12c). The resulting [*Tr*AA9A]_50_ was indeed about five times lower compared to the reaction with 5.0 nM HRP (Table 1) while the (*k*_cat_/${K_{{\rm{mH}}_2{\rm{O}}_2}}$)^app^ values obtained using two different [HRP] were within error limits of each other (Table 1).

The observation that variants of our kinetic approach, varying in both the type and concentration of the reference enzyme, lead to similar kinetic values adds confidence to this approach and indicates the plausibility of the assumptions that [H_2_O_2_] ≪ ${K_{{\rm{mH}}_2{\rm{O}}_2}}$^ref^ and [H_2_O_2_] ≪ ${K_{{\rm{mH}}_2{\rm{O}}_2}}$, that needed to be made in deriving the equations. We have also estimated the maximum (i.e in the absence of competing enzyme) steady-state concentrations of H_2_O_2_ ([H_2_O_2_]_max_) for the different reference enzyme systems used in this study (Supplementary Table 1). Although in a few cases the [H_2_O_2_]_max_ values were in the same order as the apparent ${K_{{\rm{mH}}_2{\rm{O}}_2}}$ of the reference enzymes (Supplementary Table 1), the true steady-state H_2_O_2_ concentrations in the competition experiments are expected to be lower because of the presence of the competing enzyme (Supplementary Discussion, Eqs. (S9–S11)).

### Measuring the *k*_cat_/${K_{{\rm{mH}}_2{\rm{O}}_2}}$ values of a bacterial cellulose-active LPMO

**Fig. 6:**
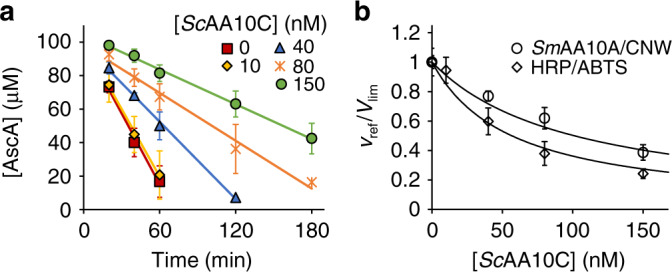
Inhibition of HRP/ABTS and *Sm*AA10A/CNW by *Sc*AA10C /Avicel. All experiments were made in Bis-Tris buffer (50 mM, pH 6.1) at 25 °C and contained, AscA (0.1 mM), Avicel (100 g L^−1^) and glucose (10 mM). **a** Progress curves for the oxidation of ABTS (0.2 mM) by HRP (1.0 nM) (measured as consumption of AscA) in the presence of *Sc*AA10C at different concentrations. Reactions were provided with GO (0.035 g L^−1^). Solid lines show linear regression of the data. The slopes of the lines correspond to the rate of the reference reaction (*v*_ref_). **b** Dependency of *v*_ref_/*V*_lim_ on [*Sc*AA10C]. The data for the HRP/ABTS reference reaction are from panel (**a**). The data for the *Sm*AA10A/CNW reference reaction are from Supplementary Fig. 13. Solid lines show nonlinear regression of the data according to Eq. (5). Data are presented as average values (*n* = 3, independent experiments) and error bars show SD. Source data are provided as a Source Data file.

The apparent *k*_cat_/${K_{{\rm{mH}}_2{\rm{O}}_2}}$ for *Sm*AA10A acting on insoluble chitin (1.5 μM^−1^ s^−1^; Supplementary Table 1) and the previously determined true *k*_cat_/${K_{{\rm{mH}}_2{\rm{O}}_2}}$ (2.4 μM^−1^ s^−1^)^14^ are higher compared to the values determined for fungal AA9 LPMOs acting on cellulose (Table 1). To get an indication as to whether this difference reflects differences between LPMO families or between substrates, we also determined the apparent *k*_cat_/${K_{{\rm{mH}}_2{\rm{O}}_2}}$ of a cellulose-active family AA10 LPMO from the bacterium *S. coelicolor* (*Sc*AA10C), using Avicel (100 g L^−1^) as substrate. GO with glucose was used as a source of H_2_O_2_ and the apparent *k*_cat_/${K_{{\rm{mH}}_2{\rm{O}}_2}}$ was measured using two reference reactions, HRP/ABTS (Fig. 6) and *Sm*AA10A/CNW (Supplementary Fig. 13). The apparent *k*_cat_/${K_{{\rm{mH}}_2{\rm{O}}_2}}$ values for *Sc*AA10C obtained using two very different reference reactions were within error limits to each other (0.13–0.15 μM^−1^ s^−1^; Table 1) and more similar to the values obtained with the cellulose-active fungal AA9 LPMOs than the bacterial chitin-active AA10 LPMO. These values were derived from measurements at only one Avicel concentration and are therefore apparent values. Since 100 g L^−1^ is the highest applicable Avicel concentration, the resulting apparent *k*_cat_/${K_{{\rm{mH}}_2{\rm{O}}_2}}$ values are close to upper limit achievable in practice.

## Discussion

Although the nature of the natural co-substrate of LPMOs, O_2_, or H_2_O_2_, remains a subject of some debate, it is clear from existing data, that the peroxygenase reaction is orders of magnitude faster than the monooxygenase reaction and thus, likely, more relevant under most conditions. Interestingly, studies on a totally different enzyme done prior to the discovery of the peroxygenase activity of LPMOs have shown that a non-heme-iron epoxidase originally thought to be an oxidase in fact is a peroxidase and, as such, orders of magnitude faster than previously thought^32^. The implications of these discoveries for LPMO activity on cellulose have remained unclear, due to a lack of kinetic studies, and this adds to maintaining doubt regarding the nature of the co-substrate and possible differences between LPMOs belonging to different families. Here, we present a kinetic assessment of the peroxygenase reaction carried out by LPMOs belonging to multiple LPMO families, acting on various substrates. The data show that, in terms of H_2_O_2_ consumption, the kinetic properties of these LPMOs are similar to those of known peroxygenases. Clearly, H_2_O_2_-driven cellulose degradation by LPMOs is chemically and biologically meaningful, as discussed further below.

Measurement of the kinetic parameters of LPMOs is complicated by numerous possible LPMO-dependent and -independent cross-reactivities of the reductant and oxidant^12^. These interfering side reactions cannot be “taken away” but their contribution can be minimized by choosing appropriate experimental conditions, including appropriate control reactions, as we have done here. The use of the GO/glucose reaction as a source of H_2_O_2_ gave high rates of H_2_O_2_ production (high *V*_lim_) that made the contribution of other possible H_2_O_2_ producing routes, like the reaction between O_2_ and reductant or the uncoupled reaction between O_2_ and the LPMO, insignificant. Control experiments with fixed initial H_2_O_2_ loads suggested that the substrates of the LPMOs of interest did not significantly inhibit the reference enzymes *Sm*AA10A (Supplementary Fig. 3) and HRP (Supplementary Fig. 7). However, the HRP substrates, ABTS and AR, seemed to be somewhat inhibitory for *Sm*AA10A (Supplementary Fig. 3). As alluded to above, not all possible interactions between reaction components and the various enzymes used could be experimentally assessed. Most importantly, however, the similar *k*_cat_/${K_{{\rm{mH}}_2{\rm{O}}_2}}$ values for the same LPMO of interest obtained using very different reference reactions like *Sm*AA10A/CNW, HRP/AR, and HRP/ABTS (Table 1), strongly suggest that, under our experimental conditions, the LPMOs of interest were not inhibited by components of the reference reactions. In cases where inhibition of LPMOs by HRP substrates is suspected, and when alternative reference reactions (like the *Sm*AA10A/CNW reaction used in this study) are not available, we suggest to perform measurements with varying concentrations of HRP substrate as control reactions. In principle, the competition experiments described here are applicable for LPMOs acting on a variety of substrates, such as a variety of cellulosic substrates, hemicelluloses, starch, and cellulose–hemicellulose composites. For LPMOs acting on soluble substrates and generating only soluble products that are easy to quantify, the use more typical standard enzyme provides a simpler alternative. As for lignin-rich biomass, we expect complications, in part because of the redox and radical-formation properties of lignin, which may lead to interference with the HRP assay in multiple ways. While the (less generally applicable) reference reaction with the chitin-active LPMO could likely still be used, another major complication comes from the reactivity of lignin with H_2_O_2_^40^.

Unlike the *k*_cat_/${K_{{\rm{mH}}_2{\rm{O}}_2}}$ values, the half-saturating substrate concentrations ([S]_0.5_ in Eq. (7)) obtained for the same LPMO of interest using different reference reactions differed significantly and these values must be treated with caution. Accurate determination of [S]_0.5_ implies measurements at low, sub-saturating substrate concentrations. The concentration of substrate-free LPMO increases with decreasing substrate concentration, increasing thereby the possible contribution of side reactions like uncoupled reduction of O_2_ or H_2_O_2_, which both rely on polysaccharide-free reduced LPMO^18^. Furthermore, the applicability of the Michaelis–Menten theory for heterogeneous interfacial catalysis may break down at low concentrations of polysaccharide^41^. Therefore, the competition experiments are best suited for high concentrations of LPMO substrate, and these conditions are also more relevant regarding the industrial application of LPMOs.

Because of the commercial availability of the components, the HRP based reference reaction is of particular interest. HRP is active on many different substrates and in many cases the initial product is a radical that is converted into a detectable product in a HRP-independent reaction between free radicals^42^. Such reactions may also lead to the formation of different side products and decrease the signal intensity per molecule of H_2_O_2_ consumed. As a consequence, the resulting progress curves are often non-linear, complicating accurate measurement of initial rates. For example, we noted the dependency of the apparent absorbance yield of the HRP/AR reaction on [AscA] (Supplementary Fig. 8), which is clearly due to side reactions. Although we obtained meaningful results with the HRP/AR reference reaction, to overcome the complications related to side reactions, we used ABTS as the HRP substrate and followed its oxidation by the measuring the consumption of AscA^37,42^. This setup is sensitive for enzyme independent oxidation of AscA by O_2_^43^ and H_2_O_2_^37^. These reactions are, however, slow, as long as care is taken in removing trace amounts of divalent metal ions that catalyze AscA oxidation^44,45^, as we did in this study by treating insoluble substrates with EDTA and solutions with Chelex resin (Supplementary Fig. 14).

HRP has been shown to have AscA oxidizing^37^ as well as catalase-like^46^ activity but the *k*_cat_/*K*_m_ values of these reactions are more than three orders of magnitude lower than that for the H_2_O_2_-driven oxidation of ABTS^37^. Therefore, HRP-catalyzed oxidation of AscA is unlikely in the presence of ABTS. Possible depletion of AscA by LPMO can be significant but, as we show here (Supplementary Fig. 11), only at low concentrations of the LPMO substrate. Considering the nature of the peroxygenase reaction, with a priming reduction of the LPMO^13,21,22^, higher concentrations of substrate will reduce the amount of free LPMO in solution, which reduces the rate of LPMO oxidation, which again reduces the need for re-reduction by AscA.

H_2_O_2_-active enzymes like catalases and peroxidases have been added to LPMO reactions in previous studies. In some studies, the competing enzymes were added to reduce possible inactivation of (any) enzymes by H_2_O_2_^28,47,48^, whereas other studies aimed at showing that the main co-substrate in LPMO catalysis was either O_2_^15,26,49^ or H_2_O_2_^13,29^. Complete inhibition of LPMOs by HRP has been interpreted as the dependency of LPMOs on H_2_O_2_^13,29^, whereas lack of such inhibition under certain reaction conditions has been taken to show that LPMOs use O_2_ as co-substrate^15,26^. While the present data clearly show that multiple LPMOs use H_2_O_2_ effectively, the results presented here do not rule out the possible use of O_2_ as co-substrate. Similar conclusions were reached in recent studies on the reoxidation of LPMOs by H_2_O_2_ in the absence of substrate^18,20^. We also note that competition experiments do not enable unequivocal discrimination between the use of H_2_O_2_ in LPMO-catalyzed polysaccharide peroxygenase and reductant peroxidase reactions. However, apparent *k*_cat_/${K_{{\rm{mH}}_2{\rm{O}}_2}}$ values in the order of 10^3^–10^4^ M^−1^ s^−1^ reported for the reductant peroxidase activity of *Nc*AA9C^16,50^, *Sm*AA10A^18^, and *Tr*AA9A^20^ are lower than the *k*_cat_/${K_{{\rm{mH}}_2{\rm{O}}_2}}$ values described here (Table 1). While a contribution of reductant peroxidase activity cannot be excluded at low substrate concentrations, the clear correlation of increasing apparent *k*_cat_/${K_{{\rm{mH}}_2{\rm{O}}_2}}$ with increasing substrate concentration (Table 1, Fig. 4) shows that the *k*_cat_/${K_{{\rm{mH}}_2{\rm{O}}_2}}$ values reported here represent peroxygenase activity on a cellulosic substrate.

The measured *k*_cat_/${K_{{\rm{mH}}_2{\rm{O}}_2}}$ values in the order of 10^5^–10^6^ M^−1^ s^−1^ (Table 1) place LPMOs firmly among other well studied peroxidases and peroxygenases, kinetically spoken and suggest that LPMOs are competitive with other H_2_O_2_ consuming enzymes that may be present in their natural environments. The highest *k*_cat_/${K_{{\rm{mH}}_2{\rm{O}}_2}}$ values, in the order of 10^6^–10^7^ M^−1^ s^−1^, have been reported for manganese peroxidases^51–54^, whereas reported *k*_cat_/${K_{{\rm{mH}}_2{\rm{O}}_2}}$ values for lignin peroxidases^55,56^ and DyP peroxidases^57,58^ usually are below 10^6^ M^−1^ s^−1^. Regarding peroxygenases, a *k*_cat_/${K_{{\rm{mH}}_2{\rm{O}}_2}}$ value in the order of 10^6^ M^−1^ s^−1^ has been reported for an unspecific peroxygenase from *Agrocybe aegerita*^59^. Bacterial and fungal catalases have also *k*_cat_/${K_{{\rm{mH}}_2{\rm{O}}_2}}$ values in the order of 10^6^ M^−1^ s^−1^ ^60^.

Considering that H_2_O_2_ is a double-edge sword, being crucial for peroxidase/peroxygenase activities but also potentially dangerous, Nature has likely evolved enzymatic systems able to function efficiently at relatively low, harmless H_2_O_2_ concentrations. In these conditions, the *k*_cat_/${K_{{\rm{mH}}_2{\rm{O}}_2}}$ is a reliable metrics to describe LPMOs and to assess their competitiveness with other H_2_O_2_ consuming enzymes in their native environments. The similar catalytic efficiencies of fungal H_2_O_2_ consuming enzymes could be rationalized by assuming that these enzymes share the same catalytic chemistries, as could be the case for the many known heme-iron enzymes. The fact that LPMOs, which are mono-copper, and otherwise co-factor free enzymes, display similar catalytic efficiencies may reflect co-adaptation of enzymatic systems to a common redox environment.

In conclusion, we have developed a method to kinetically assess the cellulolytic peroxygenase reaction and we show, for multiple LPMOs and using multiple reference enzymes that the kinetic values for this reaction are similar to those observed for other peroxygenases and peroxidases. The method used requires great care, for example due to the multiple possible side reactions, but only requires readily available chemicals and equipment. The fact that we obtained similar results when assessing the same LPMO with different reference enzymes shows that the method is robust and adds credibility to the obtained kinetic values. Most importantly, while biologically or industrially relevant use of O_2_ in LPMO catalysis cannot be excluded, the present data confirm that the peroxygenase mechanism is fast and relevant for LPMOs acting on insoluble cellulose.

## Methods

### Substrates and reagents

*N*-acetyl-3,7-dihydroxyphenoxazine (AR, Amplex Red or Ampliflu Red), 2,2′-azino-bis(3-ethylbenzothiazoline-6-sulfonic acid) diammonium salt (ABTS, Lot # SLBT0759), 2,2′-bis(hydroxymethyl)-2,2′,2′′-nitrilotriethanol (Bis–Tris, Lot # SLBX0598), Tris-(hydrohymethyl)-aminomethane (Tris), l-ascorbic acid (AscA, Lot # SLBM0850V), d-glucose, ethylene-diamine-tetraacetate disodium salt dihydrate (EDTA), and potassium persulfate (Lot # MKCC7933) were from Sigma-Aldrich. Chelex^®^ 100 resin (50–100 mesh, sodium form) was from Bio-Rad. 1,4-β-d-cellopentaose (Glc_5_, Lot # 121205) was from Megazyme. The H_2_O_2_ stock solution (Lot# SZBG2070) was from Honeywell. Microcrystalline cellulose (Avicel) was from Fluka. CNWs with specific radioactivity of 4.18 × 10^6^ dpm mg^−1^ were prepared from α-chitin of crab shells exactly as described in Kuusk et al.^61^. The water was Milli-Q ultrapure water that had been passed through a column with Chelex^®^ 100 resin. Stock solutions of buffers, glucose, and Glc_5_ were kept over beds of Chelex^®^ 100 resin. To remove metal ions, CNWs and Avicel were incubated with 10 mM EDTA in 10 mM Tris pH 8.0, overnight. After that, the EDTA was removed by thorough washing with water using repetitive centrifugation and re-suspension steps. The stock solutions of CNWs and Avicel were kept in water at 4 °C. Dilutions of the commercial H_2_O_2_ stock solution (30 wt%, 9.8 M) were prepared in water, directly before use. AscA (50 mM in water) was kept as frozen aliquots at −18 °C and aliquots were melted directly before use.

### Enzymes

*Sm*AA10A and *Tr*AA9A were produced and purified as previously described in Vaaje-Kolstad et al.^1^ and Kont et al.^29^, respectively. *Nc*AA9C and *Sc*AA10C were produced and purified as previously described in Kittl et al.^23^ and Forsberg et al.^62^, respectively. *Tr*AA9A, *Nc*AA9C, and *Sc*AA10C used in this study are full-length enzymes composing of an LPMO domain connected to a carbohydrate binding module via a linker peptide whereas *Sm*AA10A is a single-domain enzyme. Purified LPMOs were saturated with copper by overnight incubation with excess CuSO_4_. The unbound copper was removed by extensive washing with sodium acetate buffer (50 mM, pH 5.0) using an Amicon ultracentrifugation device equipped with a 5 kDa cut-off membrane. The concentrations of Cu-saturated LPMOs were determined by measuring the absorbance at 280 nm using theoretical molar extinction coefficients of 35,200, 54,360, 46,910, and 75,775 M^−1^ cm^−1^ for *Sm*AA10A, *Tr*AA9A, *Nc*AA9C, and *Sc*AA10C, respectively.

GO from *Aspergillus niger* (GO, Sigma G-6125) and HRP (Sigma P8375) were used as purchased. The concentration of HRP was determined by measuring the absorbance at 403 nm using a molar extinction coefficient of 102,000 M^−1^ cm^−1^^63^. The GO was dosed on weight basis. The stock solutions of LPMOs and HRP were kept in sodium acetate (50 mM, pH 5.0) at 4 °C. The stock solution of GO, in sodium acetate (50 mM, pH 5.0), was made daily before the use.

### Competition experiments with *Sm*AA10A/CNW

All experiments were made in Bis–Tris buffer (50 mM, pH 6.1) at 25 °C in 1.5 mL polypropylene micro-centrifuge tubes. CNWs (1.0 g L^−1^) were mixed with glucose (10 mM) and Glc_5_ (0–2.0 mM) or Avicel (0–100 g L^−1^) followed by the addition of *Sm*AA10A and the LPMO of interest. Finally, the mixtures were supplied with AscA (0.1 mM) and reactions were started by the addition of GO (0.03 g L^−1^). Stirring was omitted (within the time-frame of the experiment the CNWs form stable suspensions even in the presence of Avicel) but the suspension was mixed using a pipet before withdrawing samples. At pre-defined times, 0.1 mL aliquots were withdrawn and added to the 25 µL of 1.0 M NaOH to stop the reaction. Non-labeled CNWs (3 g L^−1^) in 0.2 M NaOH were added to improve the sedimentation of the CNWs during centrifugation (5 min, 10^4^ × *g*)^61^. The concentration of ^14^C-labeled soluble products was calculated based on the radioactivity in the supernatant and was expressed in *N*-acetylglucosamine equivalents (NAG_eq_) as described in detail in Kuusk et al.^14^.

In the case of the control experiment assessing competition between *Sm*AA10A and HRP, the ABTS (0.2 mM) was added to CNWs (1.0 g L^−1^) followed by the addition of *Sm*AA10A (42 nM) and HRP (between 0 and 100 nM). The reaction was started by the addition of AscA (1.0 mM). The reactions were stopped and products were quantified as described above.

In the control experiments done to assess possible inhibition of *Sm*AA10A by various reaction components, the reactions were provided with CNWs (1.0 g L^−1^), AscA (0.1 mM), H_2_O_2_ (20 µM), and potential inhibitors (Avicel, Glc_5_, ABTS, or AR). Here the reactions were started by the addition of H_2_O_2_. The reactions were stopped and products were quantified as described above.

### Competition experiments with HRP/Amplex Red

All experiments were made in Bis–Tris buffer (50 mM, pH 6.1) at 25 °C. Glucose (10 mM) was mixed with Glc_5_ (0–1.0 mM), Amplex Red (0.2 mM), HRP (5.0 nM) and *Nc*AA9C (0–200 nM). Finally, the mixtures were supplied with AscA (0.1 mM) and reactions were started by the addition of GO (0.15 g L^−1^). Oxidation of AR was followed in the spectrophotometer cuvette by measuring the absorbance at 570 nm in time.

### Competition experiments with HRP/ABTS

All experiments were made in Bis–Tris buffer (50 mM, pH 6.1) at 25 °C in glass test-tubes. The reactions were agitated by stirring to avoid sedimentation of Avicel. Glucose (10 mM) was mixed with Avicel (0–100 g L^−1^), ABTS (0.2 mM), HRP and the LPMO of interest. Finally, the mixtures were supplied with AscA (0.1 mM) and reactions were started by the addition of GO (0.035 g L^−1^). At selected times (ranging from 20 to 180 min depending on the rate of the reaction) 0.25 mL aliquots were withdrawn and Avicel was pelleted using centrifugation (1 min, 10^4^×*g*). Then, 0.1 mL of the supernatant was added to 0.9 mL of an appropriately diluted stock solution of ABTS/ABTS^•+^ in water (the final concentration of ABTS^•+^ in the cuvette was approximately 30 µM). Totally, 10–20 s after mixing of the reagents, the absorbance at 420 was recorded using a spectrophotometer. The concentration of AscA was found from the decrease in absorbance using *ε*_420_ = 0.032 µM^−1^ cm^−1^ for ABTS^•+^ and a stoichiometry of 2ABTS^•+^/AscA^37^. The starting value of the absorbance (used in calculation of the AscA dependent decrease in absorbance) was measured exactly as described above but the supernatant was replaced with 0.1 mL of 50 mM Bis–Tris (pH 6.1). We note that 50 mM Bis–Tris buffer in the cuvette caused a time dependent decrease in the absorbance at 420 nm suggesting interference by buffer components. Such interference was not observed when using 5.0 mM Bis–Tris in the cuvette, which is the condition that was used throughout this study.

The ABTS/ABTS^•+^ stock solution was made by incubating ABTS (2.0 mM) with potassium persulfate (0.5 mM) in water, overnight, in the dark. Because of the stoichiometry of 2ABTS^•+^/potassium persulfate, the oxidation of ABTS results in an equimolar (1.0 mM/1.0 mM) mixture of ABTS/ABTS^•+^. When kept in the dark at 4 °C, the ABTS/ABTS^•+^ stock solution was stable for at least one week.

### Measurement of the kinetic parameters for HRP

All experiments were made in Bis-Tris buffer (50 mM, pH 6.1) at 25 °C. HRP (1.0 nM) was added to the H_2_O_2_ solution, and the reactions were started by the addition of ABTS. Oxidation of ABTS was followed by measuring the absorbance at 420 nM and the concentration of ABTS^•+^ was calculated using *ε*_420_ = 0.032 µM^−1^ cm^−1^. The rate of H_2_O_2_ depletion was found from the rate of ABTS^•+^ formation using a stoichiometry of 2:1 for ABTS^•+^/H_2_O_2_.

The experiments with AR substrate were made as above but the reaction was followed by the increase in the absorbance at 570 nm. The concentration of resorufin (the product of AR oxidation by HRP) was calculated using *ε*_570_ = 0.033 µM^−1^ cm^−1^ (Supplementary Fig. 8) and the rate of H_2_O_2_ depletion was found from the rate of resorufin formation using a stoichiometry of 1:1 for resorufin/H_2_O_2_^38^.

For measuring the *ε*_570_ for resorufin in the reaction of AR with H_2_O_2_, the AR (0.2 mM) was incubated with H_2_O_2_ (up to 100 µM) using a high concentration of HRP (35 nM). The reaction was completed within first few minutes and the dependency of this plateau value of the absorbance on the concentration of H_2_O_2_ was used to calculate the *ε*_570_. In one set of control experiments the *ε*_570_ the reactions were supplied with 0.1 mM AscA to check for the occurrence of side reactions.

### Reporting summary

Further information on research design is available in the Nature Research Reporting Summary linked to this article.

## Supplementary information

Supplementary Information

Reporting Summary

## Data Availability

All data are available within the article and its Supporting Information File and from the corresponding author upon reasonable request. Source data are provided with this paper.

## Source data

Source Data
