# Supplementary material for: Kinetic insights into the peroxygenase activity of cellulose-active lytic polysaccharide monooxygenases (LPMOs)
Source: Nat Commun. 2020 Nov 13;11:5786. doi: 10.1038/s41467-020-19561-8 (PMC7666214; doi:10.1038/s41467-020-19561-8)
Supplement: Supplementary file 1 — Supplementary Information [file 41467_2020_19561_MOESM1_ESM.pdf]

Supplementary material to:

Kinetic insights into the peroxygenase activity of cellulose-active lytic polysaccharide monooxygenases (LPMOs)

Riin Kont[1], Bastien Bissaro[2,3], Vincent G.H. Eijsink[2], Priit Väljamäe[1*]

[1]*Institute of Molecular and Cell Biology, University of Tartu, Estonia.*

[2]*Faculty of Chemistry, Biotechnology and Food Science, NMBU - Norwegian University of Life Sciences, Norway.*

[3]*INRAE, Aix Marseille University, UMR1163 Biodiversité et Biotechnologie Fongiques, 13009 Marseille, France.*

[*]email: priit.valjamae@ut.ee

TABLE OF CONTENTS

	Page
**Supplementary results and discussion**	
Kinetic foundation.	2
Steady state H_2O_2 concentrations in the absence and presence of the LPMO of interest.	4
**Supplementary Table**	
Table 1. Predicted steady-state concentrations of H_2O_2 ($[H_2O_2]_{max}$) in different reference reactions, with different H_2O_2 producing systems, in the absence of a (competing) enzyme of interest.	5
**Supplementary Figures**	
Figure 1. Determination of the kinetic parameters for HRP with ABTS and H_2O_2.	6
Figure 2. Dependency of the rate of chitin degradation on [*Sm*AA10A].	7
Figure 3. Chitin degradation by *Sm*AA10A in the presence of potential inhibitors.	8
Figure 4. Progress curves for the *Sm*AA10A/CNW reaction in the presence of *Tr*AA9A/Avicel.	9
Figure 5. Dependence of the rate of the reference reaction in the absence of a competing LPMO (V_{lim}) on the substrate used by the competing LPMO.	10
Figure 6. Progress curves for the *Sm*AA10A/CNW reaction in the presence of *Nc*AA9C/Glc_5.	11
Figure 7. Determination of kinetic parameters for HRP with Amplex Red (AR) and H_2O_2.	12
Figure 8. Calibration of the signal intensity (maximum change in the absorbance at 570 nm) for the reaction of HRP with Amplex Red (AR) and H_2O_2.	13
Figure 9. Progress curves for the oxidation of Amplex Red (AR) by HRP in the presence of *Nc*AA9C/Glc_5.	14
Figure 10. Effect of ascorbic acid (AscA) on detection of HRP-catalyzed oxidation of ABTS by H_2O_2.	15
Figure 11. Stability of AscA in experiments without HRP.	16
Figure 12. Progress curves for the oxidation of ABTS by HRP in the presence of *Tr*AA9A/Avicel.	17
Figure 13. Progress curves for the *Sm*AA10A/CNW reaction in the presence of *Sc*AA10C /Avicel.	18
Figure 14. The rate of chitin degradation in the absence and presence of cellopentaose (Glc_5) made using different stock solutions.	19
**Supplementary References**	20

Supplementary results and discussion

Kinetic foundation

First, we note that all rate equations that appear in the main article and supplementary material correspond to the rate equations in the absence of products (i.e. they are rate equations for the forward reaction). In all equations we refer to the parameters and substrates of the reference reaction (designated with superscript, *ref*) but the same equations apply for the enzyme of interest.

Rate equation for the ordered ternary complex mechanism with H_2O_2 being the first substrate to bind (equation S1).

$$v_{ref} = -\frac{d[H_2O_2]}{dt} = \frac{k_{cat}^{ref}[E^{ref}][H_2O_2][S^{ref}]}{K_{iH2O2}^{ref}K_{mS}^{ref} + K_{mH2O2}^{ref}[S^{ref}] + K_{mS}^{ref}[H_2O_2] + [H_2O_2][S^{ref}]} \quad \text{(S1)}$$

Equation S1 is eq. (6.8) of the textbook by Cornish-Bowden[1]. Note that equation S1 is the same as equation 1 of the main article (i.e. for the ordered ternary complex mechanism with S^{ref} being the first substrate to bind) but the parameters corresponding to S^{ref} and H_2O_2 are interchanged.

Rate equation for the random order ternary complex mechanism (equation S2).

$$v_{ref} = -\frac{d[H_2O_2]}{dt} = \frac{k_{cat}^{ref}[E^{ref}][H_2O_2][S^{ref}]}{K_{iS}^{ref}K_{mH2O2}^{ref} + K_{mH2O2}^{ref}[S^{ref}] + K_{mS}^{ref}[H_2O_2] + [H_2O_2][S^{ref}]} \quad \text{(S2)}$$

Equation S2 is eq. (6.6) of the textbook by Cornish-Bowden[1] but in the absence of products. Equation S2 assumes that all binding steps are at equilibrium (rapid equilibrium assumption). Without this assumption, the steady-state equation for the random order mechanism cannot be written in terms of K_m and K_i constants[2]. Because S^{ref} and H_2O_2 are interchangeable in the random order mechanism, the term $K_{iS}^{ref}K_{mH2O2}$ is the same as the term $K_{iH2O2}K_{mS}^{ref}$ [1]. Note that equation S2 is the same as equation 1 of the main article that corresponds to the ordered ternary complex mechanism with S^{ref} being the first substrate to bind. Because the terms $K_{iS}^{ref}K_{mH2O2}$ and $K_{iH2O2}K_{mS}^{ref}$ are equivalent, equation S2 is also the same as the equation S1 that corresponds to the ordered ternary complex mechanism with H_2O_2 being the first substrate to bind.

Simplified rate equations for conditions of low H_2O_2 concentrations, i.e $[H_2O_2] << K_{mH2O2}$.

Dividing numerators and denominators of equation 1, equation S1, and equation 2 with K_{mH2O2} results in equations S3, S4, and S5, respectively. Thus, we can write:

For the ordered ternary complex mechanism with S^{ref} being the first substrate to bind (equation S3)

$$v_{ref} = -\frac{d[H_2O_2]}{dt} = \frac{\frac{k_{cat}^{ref}}{K_{mH2O2}^{ref}}[E^{ref}][H_2O_2][S^{ref}]}{K_{iS}^{ref} + K_{mS}^{ref}\frac{[H_2O_2]}{K_{mH2O2}^{ref}} + [S^{ref}] + \frac{[H_2O_2]}{K_{mH2O2}^{ref}}[S^{ref}]} \quad \text{(S3)}$$

For the ordered ternary complex mechanism with H_2O_2 being the first substrate to bind (equation S4)

$$v_{ref} = -\frac{d[H_2O_2]}{dt} = \frac{\frac{k_{cat}^{ref}}{K_{mH2O2}^{ref}}[E^{ref}][H_2O_2][S^{ref}]}{\frac{K_{iH2O2}^{ref}}{K_{mH2O2}^{ref}}K_{mS}^{ref} + [S^{ref}] + K_{mS}^{ref}\frac{[H_2O_2]}{K_{mH2O2}^{ref}} + \frac{[H_2O_2]}{K_{mH2O2}^{ref}}[S^{ref}]} \quad \text{(S4)}$$

For the ping-pong mechanism (equation S5)

$$v_{ref} = -\frac{d[H_2O_2]}{dt} = \frac{\frac{k_{cat}^{ref}}{K_{mH2O2}^{ref}}[E^{ref}][H_2O_2][S^{ref}]}{K_{mS}^{ref}\frac{[H_2O_2]}{K_{mH2O2}^{ref}}+[S^{ref}]+\frac{[H_2O_2]}{K_{mH2O2}^{ref}}[S^{ref}]} \quad \text{(S5)}$$

In the conditions of $[H_2O_2] << K_{mH2O2}$ the $[H_2O_2]/K_{mH2O2} \approx 0$ and the terms containing $[H_2O_2]/K_{mH2O2}$ can be neglected from the denominators of equations S3 – S5. Within these constraints we have:

For the ordered ternary complex mechanism with S^{ref} being the first substrate to bind (equation S6)

$$v_{ref} = -\frac{d[H_2O_2]}{dt} \approx \frac{\frac{k_{cat}^{ref}}{K_{mH2O2}^{ref}}[E^{ref}][H_2O_2][S^{ref}]}{K_{iS}^{ref}+[S^{ref}]} \quad \text{(S6)}$$

For the ordered ternary complex mechanism with H_2O_2 being the first substrate to bind (equation S7)

$$v_{ref} = -\frac{d[H_2O_2]}{dt} \approx \frac{\frac{k_{cat}^{ref}}{K_{mH2O2}^{ref}}[E^{ref}][H_2O_2][S^{ref}]}{\frac{K_{iH2O2}^{ref}}{K_{mH2O2}^{ref}}K_{mS}^{ref}+[S^{ref}]} \quad \text{(S7)}$$

For the ping-pong mechanism (equation S8)

$$v_{ref} = -\frac{d[H_2O_2]}{dt} \approx \frac{k_{cat}^{ref}}{K_{mH2O2}^{ref}}[E^{ref}][H_2O_2] \quad \text{(S8)}$$

Since the rate equation for the random order ternary complex mechanism (within the constraints of the rapid equilibrium assumption, eq. S2) is the same as the equations for the ordered mechanism (equation 1 of the main article and equation S1), the simplified equation S6 applies also for the random order mechanism (since $K_{iH2O2}K_{mS}^{ref} = K_{iS}^{ref}K_{mH2O2}$, equation S7 also simplifies to equation S6).

The general form of the rate equations for the ordered mechanism (equations S6 and S7) is the same but the terms reflecting "apparent" binding strength of the substrate (leftmost term in the denominators of equations S6 and S7) are different. Therefore, while the k_{cat}/K_{mH2O2} values derived using competition experiments are independent (at least within the mechanisms considered here) the meaning of the half-saturating concentration of substrate (obtained by the analysis of the dependency of apparent k_{cat}/K_{mH2O2} values on substrate concentration according to equation 7 of the main article) depends on which mechanism is used by the enzyme. For this reason, we refer to the parameter that appears in leftmost term in the denominators of equations S6 and S7 as a half-saturating substrate concentration for k_{cat}/K_{mH2O2} ($[S]_{0.5}$.) in equation 7 of the main article.

Steady-state H_2O_2 concentrations in the absence and presence of the LPMO of interest

In the absence of an LPMO of interest the steady-state concentration of H_2O_2 ($[H_2O_2]_{max}$) can be calculated according to equation S9 using the data in Supplementary Table 1.

$$[H_2O_2]_{max} = \frac{V_{lim}K_m^{app,ref}}{V_{max}^{app,ref} - V_{lim}} \quad \text{(S9)}$$

In equation S9 the V_{lim} is the rate of H_2O_2 production by the reaction used for H_2O_2 supply. $V_{max}^{app,ref}$ is the apparent maximum velocity of H_2O_2 consumption by the reference reaction and $K_m^{app,ref}$ is the apparent Michaelis constant of the reference enzyme for H_2O_2.

With two H_2O_2-consuming enzymes (reference enzyme and LPMO of interest) being present, it is evident that the true steady-state concentration of H_2O_2 ($[H_2O_2]_{steady}$) is always lower than the $[H_2O_2]_{max}$ that was calculated (according to eq S9) for the reaction in which only E^{ref} is present. In our experimental system the rate of H_2O_2 consumption is given by the V_{lim} of its production. Thus, the system where both E^{ref} and E of interest are present can be described by equation S10.

$$V_{lim} = v_{ref} + v = \frac{V_{max}^{app,ref}[H_2O_2]_{steady}}{K_m^{app,ref} + [H_2O_2]_{steady}} + \frac{V_{max}^{app}[H_2O_2]_{steady}}{K_m^{app} + [H_2O_2]_{steady}} \quad \text{(S10)}$$

The values of the apparent Michaelis-Menten parameters depend on the concentration of the second substrate (e.g., cellulose) in a manner defined by the enzyme mechanism (i.e ping-pong or ternary complex) but they are constant throughout the experiment. The kinetic analyses in this study rely on the assumption that $[H_2O_2]_{steady}$ is lower than K_{mH2O2} for both enzymes. The $[H_2O_2]_{max}$ values in Supplementary Table 1 show that this may not always be the case, but we also know that, in the two enzyme reactions, $[H_2O_2]_{steady}$ is lower than $[H_2O_2]_{max}$.

As, an example, let`s consider the "worst case" (i.e., the case with the highest $[H_2O_2]_{max}$ value in supplementary Table 1, which is HRP/ABTS, 1 nM HRP). The high $[H_2O_2]_{max}$ in this example stems from the low concentration of HRP. The resulting $[H_2O_2]_{max}$ (7.5 µM) is higher than the apparent K_m (2.9 µM) of HRP for H_2O_2 (the low apparent K_m for H_2O_2 stems from the low [ABTS] relative to the K_m for ABTS, Supplementary Fig. 1). Of course, the kinetic parameters for the LPMO of interest are not known but let`s assume that it has the same parameter values as *Sm*AA10A. In this particular example, the apparent $K_m$ values for $H_2O_2$ are essentially equivalent for HRP (with 0.2 mM ABTS) and *Sm*AA10A (Supplementary Table 1; 2.8 and 2.9 µM, respectively). Let`s refer to them collectively as K_m^{eq}. In this special case $[H_2O_2]_{steady}$ can then be calculated according to equation S11.

$$[H_2O_2]_{steady} = \frac{V_{lim}K_m^{eq}}{V_{max}^{app,ref} + V_{max}^{app} - V_{lim}} \quad \text{(S11)}$$

Inserting the parameter values for the system with 1.0 nM HRP and 50 nM *Sm*AA10A (Supplementary Table 1) in equation S11 results in $[H_2O_2]_{steady} = 0.24 \pm 0.14$ µM. This value is an order of magnitude lower than the apparent K_m values for both HRP and *Sm*AA10A (Supplementary Table 1).

Supplementary Table 1. Predicted steady-state concentrations of H_2O_2 ($[H_2O_2]_{max}$) in different reference reactions, with different H_2O_2 producing systems, in the absence of a (competing) enzyme of interest

				Apparent kinetic parameters of the reference reaction[a]				
H_2O_2 supply[b]	V_{lim} (µM H_2O_2 min^{-1})[c]	Reference reaction[d]	$[E]_{ref}$ (nM)	k_{cat}^{app} (s^{-1})	K_m^{app} for H_2O_2 (µM)	k_{cat}^{app}/K_m^{app} for H_2O_2 ($µM^{-1}$ s^{-1})	V_{max} µM H_2O_2 (min^{-1})	$[H_2O_2]_{max}$ (µM)[f]
1 mM AscA autooxidation	0.038 ± 0.004	*Sm*AA10A / CNW	42	4.2 ± 0.3	2.8 ± 1.3	1.5 ± 0.7	10.6 ± 0.8	0.010 ± 0.005
GO 0.03 g L^{-1}	1.39 ± 0.13	*Sm*AA10A / CNW	50	4.2 ± 0.3	2.8 ± 1.3	1.5 ± 0.7	12.6 ± 0.9	0.35 ± 0.16
GO 0.03 g L^{-1}	0.95 ± 0.04	*Sm*AA10A / CNW	10	4.2 ± 0.3	2.8 ± 1.3	1.5 ± 0.7	2.6 ± 0.2	1.7 ± 0.8
GO 0.15 g L^{-1}	6.3 ± 0.9	HRP/AR	5.0	1084 ± 3	83 ± 7	13.4 ± 0.5	325 ± 1	1.6 ± 0.2
GO 0.035 g L^{-1}	1.24 ± 0.12	HRP/ABTS	5.0	25 ± 2.6[e]	2.9 ± 1.6[e]	7.1 ± 1.2	7.5 ± 0.8	0.57 ± 0.32
GO 0.035 g L^{-1}	1.08 ± 0.17	HRP/ABTS	1.0	25 ± 2.6[e]	2.9 ± 1.6[e]	7.1 ± 1.2	1.5 ± 0.16	7.5 ± 4.3

[a] The values of the apparent kinetic parameters for the *Sm*AA10A/CNW reference reaction were calculated using the true parameter values reported in Kuusk *et. al.* 2018[3] and a concentration of CNWs of 1.0 g L^{-1}. The values of the apparent kinetic parameters for the HRP/AR and HRP/ABTS reference reactions were measured in this study.

[b] Reactions with H_2O_2 supply by glucose oxidase (GO) also contained 10 mM glucose and 0.1 mM ascorbic acid (AscA).

[c] Maximum rates of H_2O_2 production (V_{lim}) by different H_2O_2 producing systems used in this study. For the *Sm*AA10A/CNW reference reaction, the V_{lim} was calculated from the rate of NAG_{eq} formation using a previously established stoichiometry of 4 NAG_{eq}/H_2O_2. For the HRP/AR reference reaction, the V_{lim} was calculated from the change in absorbance at 570 nm using an apparent ε_{570} of 0.011 $µM^{-1}$ cm^{-1} (Supplementary Fig. 8) and a stoichiometry of 1/1 for resorufin/H_2O_2. For the HRP/ABTS reference reaction, the V_{lim} was calculated from the rate of the consumption of AscA using a stoichiometry of 1/1 for AscA/H_2O_2. Different V_{lim} values obtained in experiments with the same concentration of GO reflect variations in activity between different GO stock solutions.

[d] In the case of the horseradish peroxidase (HRP)/ABTS reference reaction, the rate of the reference reaction was measured by measuring the rate of AscA depletion. The concentrations of the substrates in the reference reactions were 1.0 g L^{-1} for CNW and 0.2 mM for Amplex Red (AR) and ABTS.

[e] The low K_m value for H_2O_2 at [ABTS] = 0.2 mM prevented direct measurement of apparent k_{cat} and K_m values. Therefore, the apparent k_{cat} and K_m values were calculated by non-linear regression analysis of the

data in Supplementary Fig. 1 B & C, according to the hyperbolic function in the form of eq 7 of the main article.

[f] $[H_2O_2]_{max}$ was calculated according to the equation S9 using the data in Supplementary Table S1.

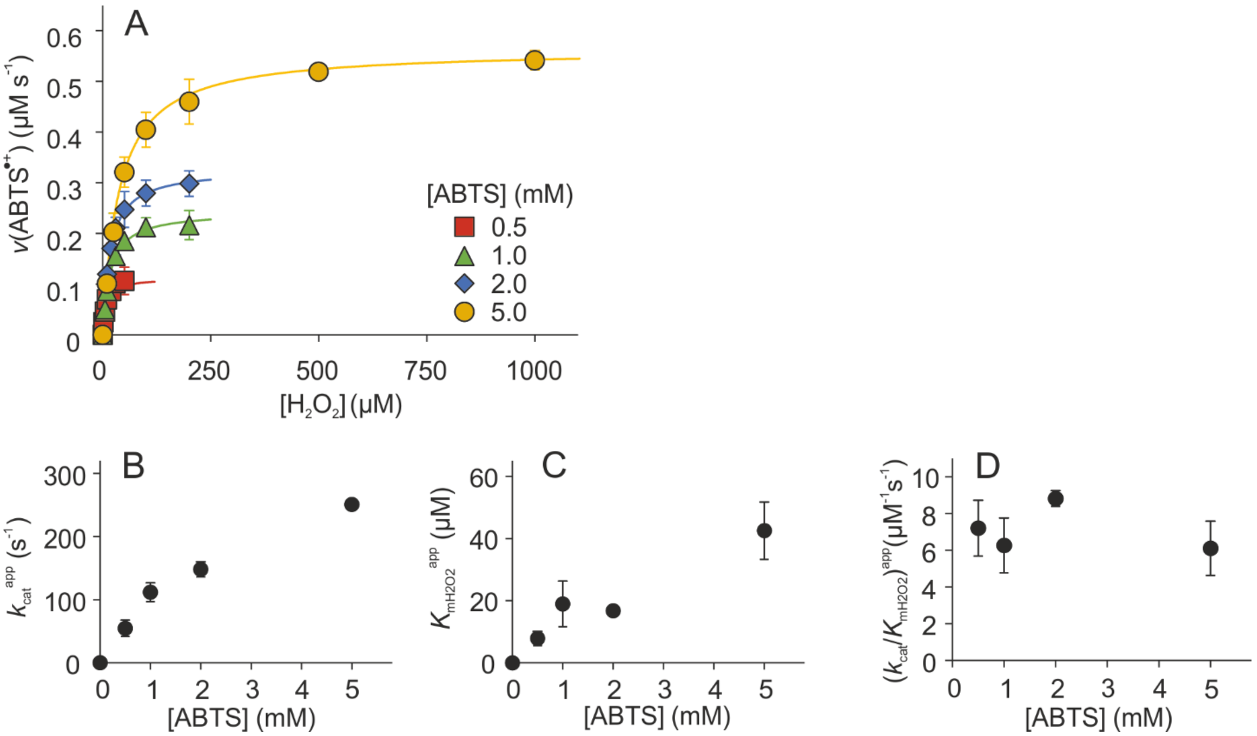

Supplementary Figure 1. Determination of the kinetic parameters for HRP with ABTS and H_2O_2. All experiments were made in Bis-Tris buffer (50 mM, pH 6.1) at 25 ºC and contained HRP (1.0 nM), ABTS, and H_2O_2. (*A*) Dependency of initial rates of ABTS oxidation on $[H_2O_2]$. Initial rates ($v_{ABTS\bullet+}$) were calculated from the change in absorbance at 420 nM after 30 s of the reaction using $\varepsilon_{420} = 0.032\ \mu M^{-1}\ cm^{-1}$ for the ABTS cation radical ($ABTS^{\bullet+}$). The concentration of ABTS is indicated in the plot. (*B* – *D*) Dependency of apparent (*B*) k_{cat}, (*C*) K_m for H_2O_2, and (*D*) k_{cat}/K_m for H_2O_2 on the concentration of ABTS. The k_{cat} represents the turnover of H_2O_2 and was calculated using the stoichiometry of $2ABTS^{\bullet+}/H_2O_2$. Error bars show *SD* derived from averaging values from three independent experiments. Since the apparent k_{cat}/K_m was independent on [ABTS], the k_{cat}/K_m for H_2O_2 used in this study ($7.1 \pm 1.2\ \mu M^{-1}\ s^{-1}$) was calculated as an average over the experiments made at different [ABTS]. Source data are provided as a Source Data file.

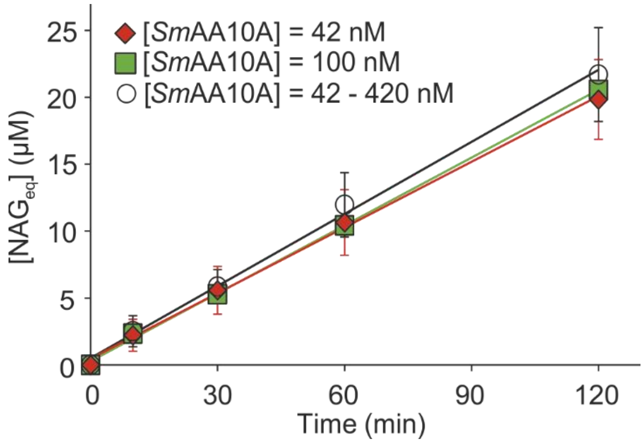

Supplementary Figure 2. Dependency of the rate of chitin degradation on [*Sm*AA10A]. All experiments were made in Bis-Tris buffer (50 mM, pH 6.1) at 25 ºC and contained CNWs (1.0 g L^{-1}), AscA (1.0 mM), and *Sm*AA10A. The formation of ^{14}C-soluble products in time is expressed in NAG equivalents (NAG_{eq})[3]. The concentration of *Sm*AA10A is indicated in the plot. The diamonds and squares show average values from experiments with 42 nM (n = 3, independent experiments; the error bars show *SD*) and 100 nM (n = 2, independent experiments) enzyme, respectively. The open circles show average values, with *SD*s as error bars, derived from four experiments, each in single parallel (except experiments with 42 nM *Sm*AA10A which were made in triplicate), with [*Sm*AA10A] = 42, 100, 200, or 420 nM in the presence of ABTS (0.2 mM). Source data are provided as a Source Data file.

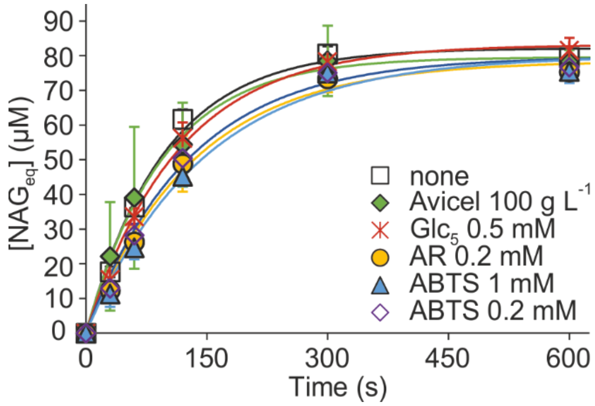

Supplementary Figure 3. Chitin degradation by *Sm*AA10A in the presence of potential inhibitors. All experiments were made in Bis-Tris buffer (50 mM, pH 6.1) at 25 ºC and contained CNWs (1.0 g L^{-1}), AscA (0.1 mM), H_2O_2 (20 µM), and *Sm*AA10A (50 nM). The formation of ^{14}C-soluble products in time is expressed in NAG equivalents (NAG_{eq}). Reactions were supplied with different potential inhibitors as indicated in the plot (see Supplementary Fig. 5 A & B for more data). Solid lines show the best fit to a single exponential function (eq. 1 in Kuusk *et. al*. 2018)[3]. Data are presented as average values (n = 3, independent experiments) and error bars show *SD*. Initial rates were calculated as in Kuusk *et. al*. 2018[3]. The initial rate without added potential inhibitors was 0.85 ± 0.06 µM NAG_{eq} s^{-1}. The initial rates in the presence of Avicel (100 g L^{-1}), Glc_5 (0.5 mM), AR (0.2 mM), ABTS (0.2 mM), and ABTS (1.0 mM) were 0.80 ± 0.31, 0.73 ± 0.09, 0.60 ± 0.12, 0.63 ± 0.09, and 0.55 ± 0.06 µM NAG_{eq} s^{-1}, respectively. Source data are provided as a Source Data file.

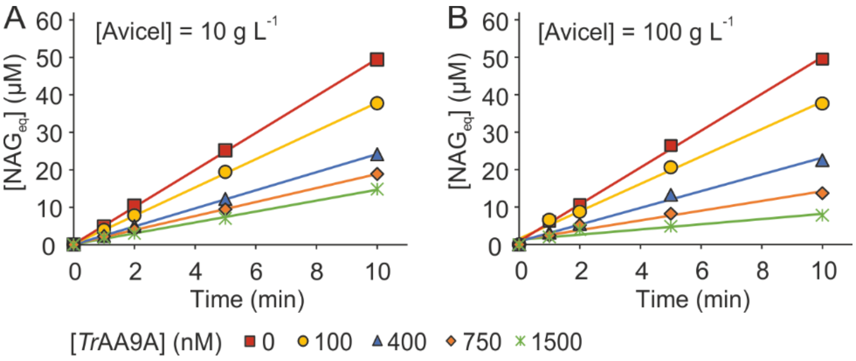

Supplementary Figure 4. Progress curves for the *Sm*AA10A/CNW reaction in the presence of *Tr*AA9A/Avicel. All experiments were made in Bis-Tris buffer (50 mM, pH 6.1) at 25 ºC and contained CNWs (1.0 g L^{-1}), AscA (0.1 mM), *Sm*AA10A (50 nM), GO (0.03 g L^{-1}), and glucose (10 mM). Progress curves of the release of ^{14}C-soluble products (in NAG_{eq}) in the presence of *Tr*AA9A and Avicel. The concentration of Avicel was (*A*) 10 g L^{-1}, or (*B*) 100 g L^{-1}. Solid lines show the linear regression of the data. Data are presented as average values (n = 2, independent experiments). Source data are provided as a Source Data file.

.

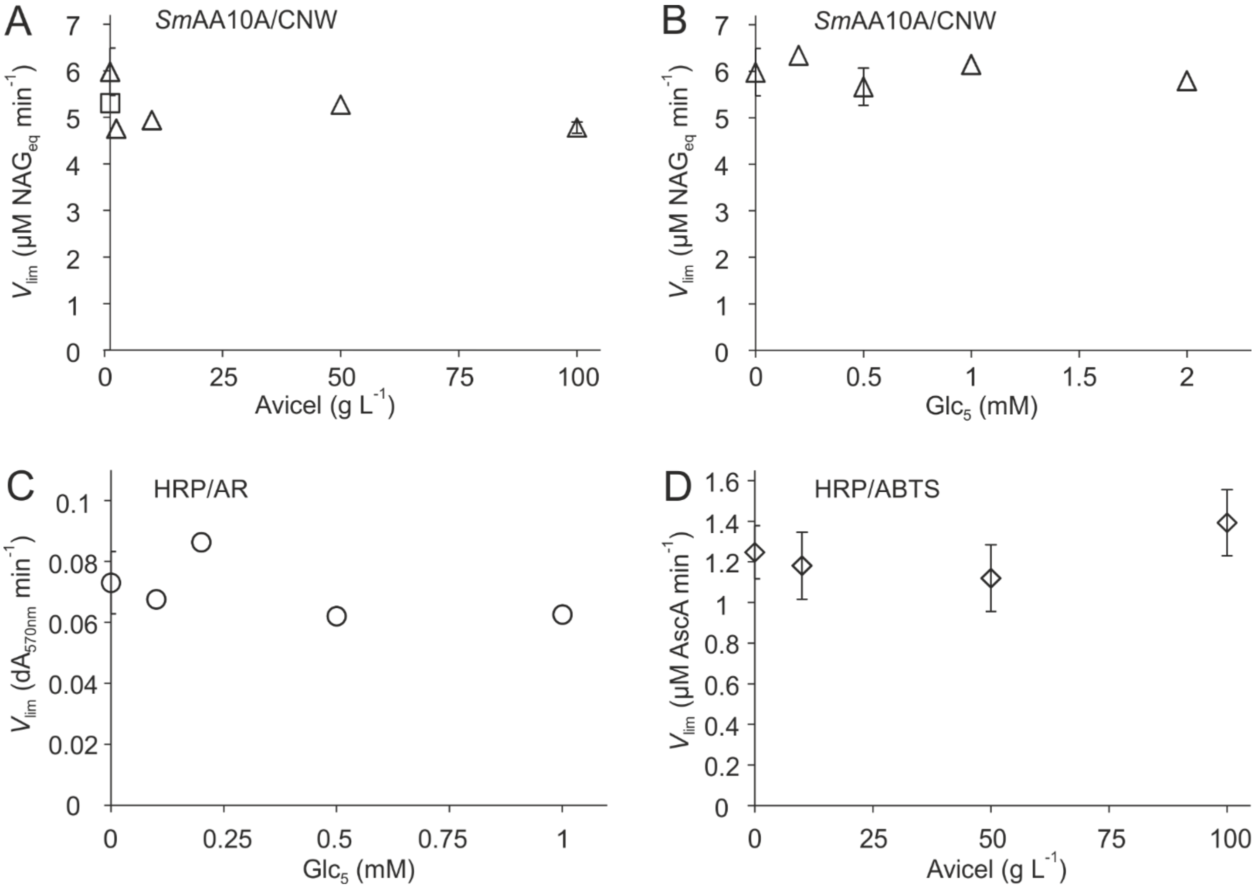

Supplementary Figure 5. Dependence of the rate of the reference reaction in the absence of a competing LPMO (V_{lim}) on the substrate used by the competing LPMO. All experiments were made in Bis-Tris buffer (50 mM, pH 6.1) at 25 ºC and contained AscA (0.1 mM), GO (0.03 g L^{-1} for panels A and B, 0.15 g L^{-1} for panel C, and 0.035 g L^{-1} for panel D), and glucose (10 mM). (*A* and *B*) Rate of the release of ^{14}C-soluble products (expressed in NAG equivalents, NAG_{eq}) from CNWs (1.0 g L^{-1}) by *Sm*AA10A (50 nM) in the presence of (*A*) Avicel (for progress curves see Fig. 3**a** and Supplementary Fig. 4) or (*B*) Glc_5 (for progress curves see Fig. 3**c** and Supplementary Fig. 6) at different concentrations. In one series in panel A the rate of *Sm*AA10A reaction was measured in the absence of Avicel but in the presence of 1500 nM *Tr*AA9A (open square). (*C*) Rate of AR (0.2 mM) oxidation (revealed as a change in absorbance at 570 nm) by HRP (5.0 nM) in the presence of Glc_5 at different concentrations (for progress curves see Fig. 5**a** and Supplementary Fig. 9). (*D*) Rate of ABTS (0.2 mM) oxidation (revealed as a disappearance of AscA) by HRP (5.0 nM) in the presence of Avicel at different concentrations (for progress curves see Fig. 5**c** and Supplementary Fig. 12 A & B). Data are presented as average values and, when present, error bars show *SD*. Data points with 2.5 g L^{-1} Avicel, and without Avicel but in the presence of *Tr*AA9A in panel A were made in single parallel. For exact number of independent experiments used in calculating average values and *SD* see the Source Data file.

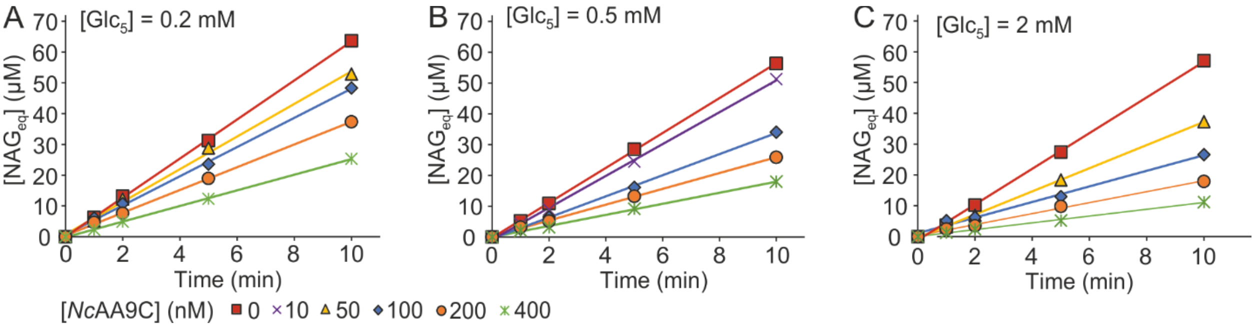

Supplementary Figure 6. Progress curves for the *Sm*AA10A/CNW reaction in the presence of *Nc*AA9C/Glc$_5$. All experiments were made in Bis-Tris buffer (50 mM, pH 6.1) at 25 ºC and contained CNWs (1.0 g L^{-1}), AscA (0.1 mM), *Sm*AA10A (50 nM), GO (0.03 g L^{-1}), and glucose (10 mM). (*A*-*C*) Progress curves of the release of ^{14}C-soluble products (in NAG$_{eq}$) in the presence of *Nc*AA9C and cellopentaose (Glc$_5$). The concentration of Glc$_5$ was (*A*) 0.2 mM, (*B*) 0.5 mM, or (*C*) 2.0 mM. Solid lines show the linear regression of the data. Data are presented as average values (n = 2, independent experiments). Source data are provided as a Source Data file.

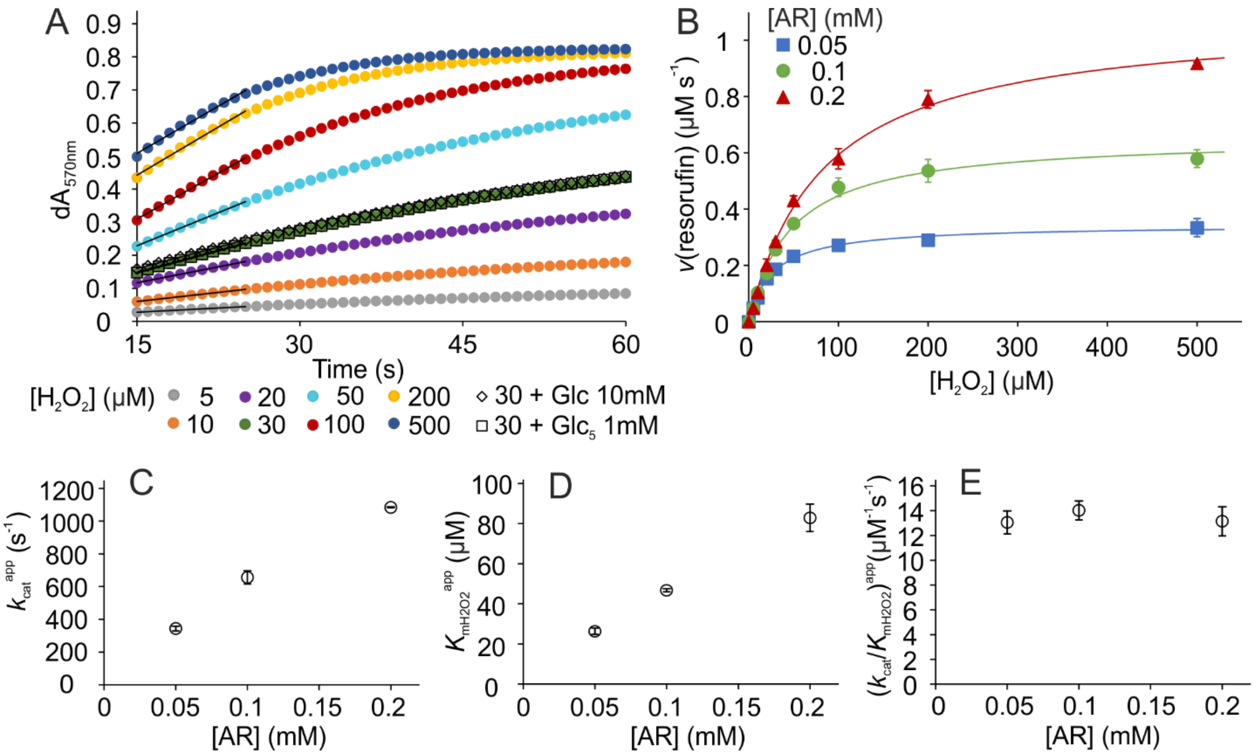

Supplementary Figure 7. Determination of kinetic parameters for HRP with Amplex Red (AR) and H_2O_2. All experiments were carried out in Bis-Tris buffer (50 mM, pH 6.1) at 25 ºC and contained HRP (1.0 nM), AR, and H_2O_2. (*A*) Representative progress curves for the oxidation of AR (0.2 mM) by HRP. The concentration of H_2O_2 is indicated in the plot. Series labeled with "30 + Glc 10 mM" and "30 + Glc_5 1 mM" contained 30 µM H_2O_2 and were supplied with 10 mM glucose and 1 mM Glc_5, respectively (both these curves are essentially identical to the curve obtained in the absence of these additions). The AR background signal (absorbance at 570 nm around 0.05) was stable within the measurement time and has been subtracted from the absorbance readings. Solid lines show best fits of linear regression analysis of the regions of the progress curves that were used to calculate initial rates. (*B*) Dependency of the initial rate of AR oxidation on [H_2O_2]. Initial rates were calculated from the change in absorbance at 570 nM, using the slopes of the solid lines shown in panel A, using an ε_{570} of 0.033 μM^{-1} cm^{-1} (Supplementary Fig. 8). The concentration of AR is indicated in the plot. Solid lines show best fits to the Michaelis-Menten equation. (*C* – *E*) Dependency of apparent (*C*) k_{cat}, (*D*) K_m for H_2O_2, and (*E*) k_{cat}/K_m for H_2O_2 on the concentration of AR. The k_{cat} represents the turnover of H_2O_2 and was calculated using a stoichiometry of one resorufin per one H_2O_2. Since the apparent k_{cat}/K_m was independent of [AR], the final k_{cat}/K_m for H_2O_2 (13.4 ± 0.5 μM^{-1} s^{-1}) used in this study was calculated as an average over the experiments made at different [AR]. Data (in *D* – *E*) are presented as average values (n = 3, independent experiments) and error bars show *SD*. Source data are provided as a Source Data file.

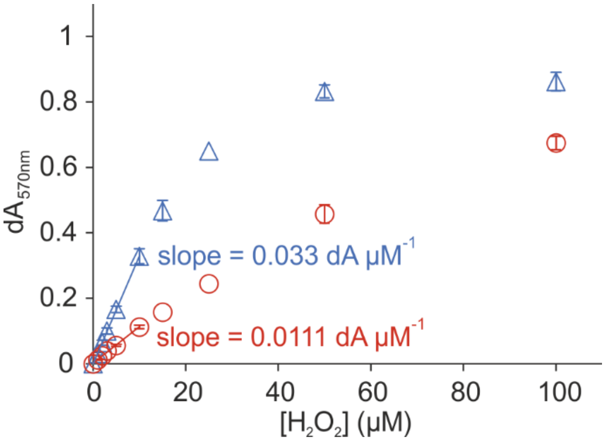

Supplementary Figure 8. Calibration of the signal intensity (maximum change in the absorbance at 570 nm) for the reaction of HRP with Amplex Red (AR) and H_2O_2. Experiments were made in Bis-Tris buffer (50 mM, pH 6.1) at 25 ºC and contained HRP (35 nM), AR (0.2 mM), and H_2O_2. The series labeled with red circles also contained 0.1 mM AscA. dA_{570nm} corresponds to the plateau value of the change in absorbance at 570 nm in time (i.e. time independent maximum change in absorbance at 570 nm, the reaction was completed within the first few minutes). Solid lines show the best fit of linear regression analysis of the data-range used in the calculation of the dA_{570nm}/H_2O_2. The lower dA_{570nm}/H_2O_2 obtained in the presence of AscA reflects the lower yield of resorufin formation from AR radicals because of the competing reduction of the AR radicals by AscA[4]. The deviation of dA_{570nm} *versus* [H_2O_2] from linearity at higher H_2O_2 concentrations can be accounted for by strong inhibition of HRP by resorufin (i.e., well known product inhibition)[5,6]. Data are presented as average values and error bars show *SD*. For the data points with error bars (n = 3, independent experiments). For the data points without error bars (n = 2, independent experiments). Source data are provided as a Source Data file.

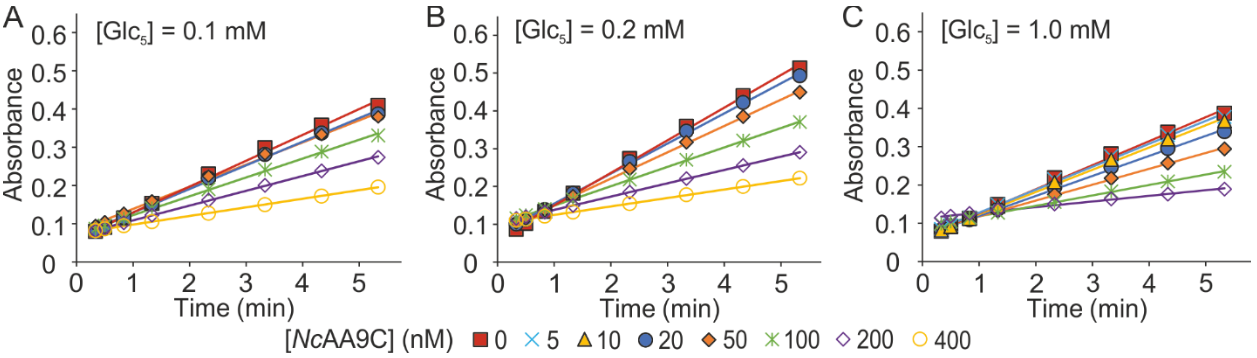

Supplementary Figure 9. Progress curves for the oxidation of Amplex Red (AR) by HRP in the presence of *Nc*AA9C/Glc$_5$. All experiments were made in Bis-Tris buffer (50 mM, pH 6.1) at 25 ºC and contained AR (0.2 mM), AscA (0.1 mM), HRP (5.0 nM), GO (0.15 g L^{-1}), and glucose (10 mM). (*A*-*C*) Progress curves for the oxidation of AR (revealed as an increase in absorbance at 570 nm) in the presence of *Nc*AA9C and cellopentaose (Glc$_5$). The concentration of Glc$_5$ was (*A*) 0.1 mM, (*B*) 0.2 mM, or (*C*) 1.0 mM. Solid lines show the linear regression of the data. Data are presented as average values (n = 2, independent experiments). Source data are provided as a Source Data file.

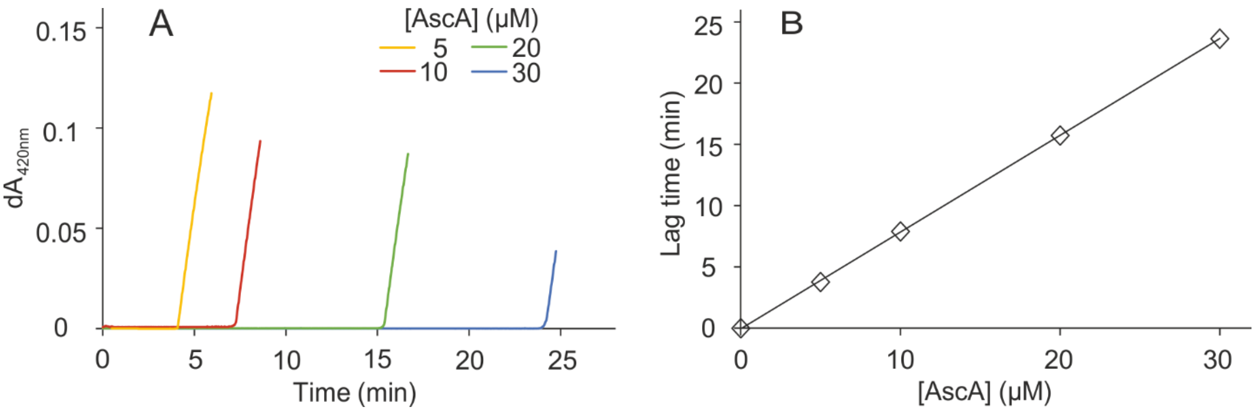

Supplementary Figure 10. Effect of ascorbic acid (AscA) on detection of HRP-catalyzed oxidation of ABTS by H_2O_2. All experiments were made in Bis-Tris buffer (50 mM, pH 6.1) at 25 ºC and contained HRP (5.0 nM), ABTS (0.2 mM), GO (0.03 g L^{-1}), and glucose (10 mM). (*A*) HRP catalyzed oxidation of ABTS (revealed as change in absorbance at 420 nm) by H_2O_2 (generated in situ by the GO reaction) in the presence of varying amounts of AscA (concentrations are indicated in the plot). Since AscA immediately reduces the ABTS cation radical ($ABTS^{•+}$) to ABTS the absorbance of $ABTS^{•+}$ is not detected before depletion of AscA. (*B*) Dependency of the lag time in $ABTS^{•+}$ detection (derived from panel A) on the concentration of AscA. The solid line represents the linear regression of the data.

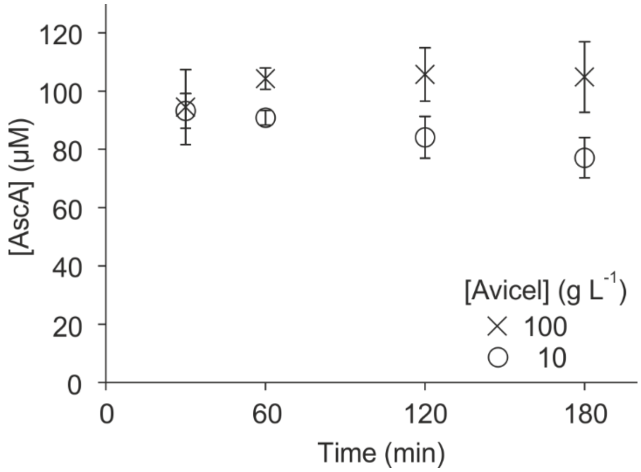

Supplementary Figure 11. Stability of AscA in experiments without HRP. Experiments were made in Bis-Tris buffer (50 mM, pH 6.1) at 25 ºC and contained, ABTS (0.2 mM), AscA (0.1 mM), GO (0.035 g L^{-1}), glucose (10 mM), *Tr*AA9A (0.4 µM), and Avicel (concentrations indicated in the plot). Data are presented as average values and error bars show *SD*. For the series with [Avicel] = 10 g L^{-1} (n = 3, independent experiments). For the series with [Avicel] = 100 g L^{-1} (n = 4, independent experiments). Source data are provided as a Source Data file.

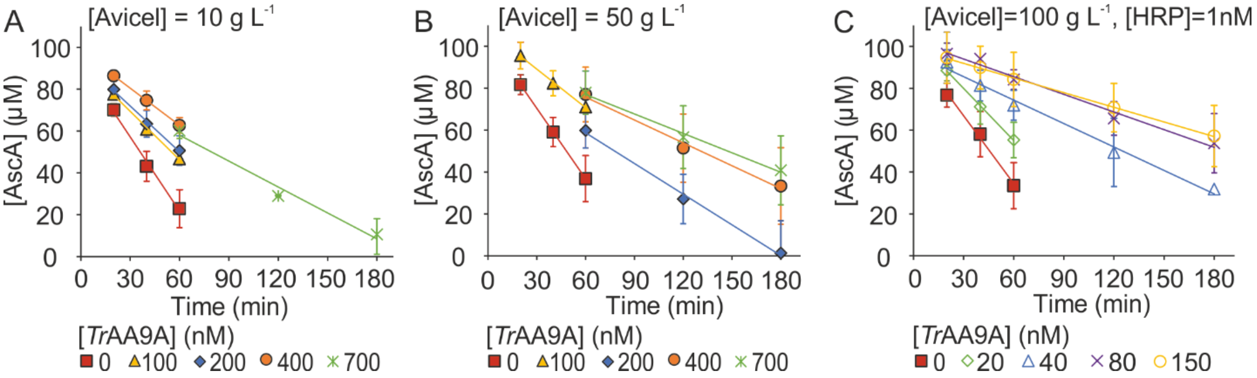

Supplementary Figure 12. Progress curves for the oxidation of ABTS by HRP in the presence of *Tr*AA9A/Avicel. All experiments were made in Bis-Tris buffer (50 mM, pH 6.1) at 25 ºC and contained ABTS (0.2 mM), AscA (0.1 mM), HRP, GO (0.035 g L^{-1}), and glucose (10 mM). (*A*-*C*) Progress curves for the oxidation of ABTS (revealed as decrease in the concentration of AscA) in the presence of *Tr*AA9A and Avicel. The concentrations of Avicel and HRP were (*A*) 10 g L^{-1} and 5.0 nM, (*B*) 50 g L^{-1} and 5.0 nM, or (*C*) 100 g L^{-1} and 1.0 nM. Solid lines show the linear regression of the data. Data are presented as average values and, when present, error bars show *SD*. For exact number of independent experiments used in calculating average values and *SD* see the Source Data file.

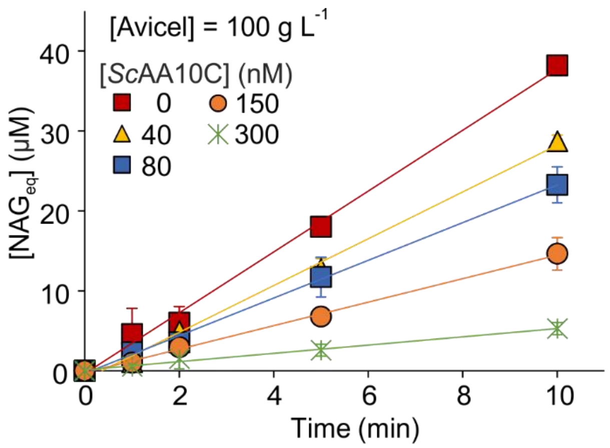

Supplementary Figure 13. Progress curves for the *Sm*AA10A/CNW reaction in the presence of *Sc*AA10C /Avicel. All experiments were made in Bis-Tris buffer (50 mM, pH 6.1) at 25 ºC and contained CNWs (1.0 g L^{-1}), AscA (0.1 mM), *Sm*AA10A (10 nM), GO (0.03 g L^{-1}), glucose (10 mM), Avicel 100 g L^{-1} and *Sc*AA10C at different concentrations. Progress curves show the release of ^{14}C-soluble products (in NAG_{eq}). Solid lines show the linear regression of the data. Data are presented as average values (n = 3, independent experiments) and error bars show *SD*. Source data are provided as a Source Data file.

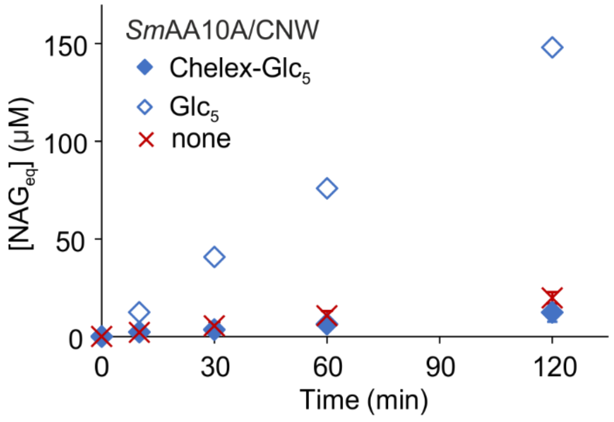

Supplementary Figure 14. The rate of chitin degradation in the absence and presence of cellopentaose (Glc_5) made using different stock solutions. All experiments were made in Bis-Tris buffer (50 mM, pH 6.1) at 25 ºC and contained CNWs (1.0 g L^{-1}), AscA (1.0 mM), and *Sm*AA10A (42 nM). The formation of ^{14}C-soluble products is expressed in NAG equivalents (NAG_{eq}). When present, the concentration of Glc_5 was 0.5 mM. The series denoted "Glc_5" was obtained using a Glc_5 preparation made by dissolving commercially available Glc_5 powder (Megazyme, Bray, Ireland) in ultrapure Trace SELECT water (Sigma Aldrich). The series denoted "$Glc_{5(Chelex)}$" was obtained using the same stock solution of Glc_5 after it had been treated with the Chelex resin to remove bivalent metal ions. Experiments with Glc_5 were done only once. For the experiments without Glc_5 and "$Glc_{5(Chelex)}$" data are presented as average values (n = 3, independent experiments) and error bars show *SD*. Source data are provided as a Source Data file.

Supplementary References

1. Cornish-Bowden, A. (1999) *Fundamentals of enzyme kinetics*, Portland Press Ltd. London. U.K.
2. Banta, S., and Wheeldon, I. (2020) Theory-based development of performance metrics for comparing multireactant enzymes. *ACS. Catal.* **10**, 1123-1132
3. Kuusk, S., Bissaro, B., Kuusk, P., Forsberg, Z., Eijsink, V. G. H., Sørlie, M., and Väljamäe, P. (2018) Kinetics of H_2O_2-driven degradation of chitin by a bacterial lytic polysaccharide monooxygenase. *J. Biol. Chem.* **293**, 523-531
4. Rodrigues, J. V, and Gomes, C. M. (2010) Enhanced superoxide and hydrogen peroxide detection in biological assays. *Free Rad. Biol. Med.* **49**, 61-66
5. Piwonski, H. M., Goomanovsky, M., Bensimon, D., Horovitz, A., and Haran, G. (2012) Allosteric inhibition of individual enzyme molecules trapped in lipid vesicles. *Proc. Natl. Acad. Sci. U.S.A.* **109**, E1437-E1443
6. Gao, Y., Liu, X., Sun, L., Xu, Y., Yang, S., Fan, C, and Li, D. (2019) Alleviated inhibition of single enzyme in confined and crowded environment. *J. Phys. Chem. Lett.* **10**, 82-89
